# Dendritic cells: understanding ontogeny, subsets, functions, and their clinical applications

**DOI:** 10.1186/s43556-025-00300-8

**Published:** 2025-09-08

**Authors:** Wenhao Li, Chenyu Yu, Xujian Zhang, Yunshen Gu, Xiaobo He, Rongrong Xu, Jia Xu, Ganjun Yu, Yanfeng Wu

**Affiliations:** 1 College of Basic Medical Sciences, Naval Medical University, Shanghai, 200433 China; 2 National Key Laboratory of Immunity and Inflammation & Institute of Immunology, College of Basic Medical Sciences, Naval Medical University, Shanghai, 200433 China

**Keywords:** Dendritic cells, Acetyl-CoA, Metabolic reprogramming, Tumor microenvironment, Immunotherapy, Cancer therapy

## Abstract

Dendritic cells (DCs) play a central role in coordinating immune responses by linking innate and adaptive immunity through their exceptional antigen-presenting capabilities. Recent studies reveal that metabolic reprogramming—especially pathways involving acetyl-coenzyme A (acetyl-CoA)—critically influences DC function in both physiological and pathological contexts. This review consolidates current knowledge on how environmental factors, tumor-derived signals, and intrinsic metabolic pathways collectively regulate DC development, subset differentiation, and functional adaptability. Acetyl-CoA emerges as a dual-function metabolite, serving not only as an energy carrier but also as an epigenetic regulator that controls DC fate via lipid biosynthesis, mitochondrial metabolism, and chromatin modification. In the tumor microenvironment (TME), DCs may experience immune suppression polarization and insufficient T cell activation due to disrupted acetyl-CoA related metabolic pathways. While existing DC-based therapies remain constrained by TME-induced metabolic limitations, emerging approaches that restore acetyl-CoA related metabolic pathways balance show enhanced antitumor efficacy. The review further examines distinct metabolic adaptations among DC subsets and their relevance to autoimmune diseases, infectious immunity, and transplant outcomes. By integrating current research on targeting DC metabolic targets, we outline strategies for developing immunotherapies that target DC metabolic flexibility. Remaining hurdles include tailoring interventions to specific subsets, refining metabolic manipulation techniques, and addressing TME heterogeneity through combination therapies. These findings position acetyl-CoA as a key therapeutic target for recalibrating immunometabolism circuits, with significant implications for DC-focused cancer treatment.

## Introduction

Dendritic cells (DCs), first identified by Ralph Steinman in 1973 as the "professional sentinels" of the immune system, serve as the critical bridge between innate and adaptive immunity by orchestrating immune surveillance, antigen presentation, and T cell priming. Over decades, their developmental ontogeny, subset specialization (e.g., cDC1, cDC2, pDC), and functional plasticity have been extensively characterized, revealing their indispensable roles in pathogen clearance, autoimmune regulation, and antitumor immunity [1, 2]. However, the tumor microenvironment (TME) imposes profound metabolic and functional constraints on DCs, leading to impaired antigen presentation, disrupted migration, and tolerogenic polarization—key barriers to effective cancer immunotherapy [3]. Emerging evidence highlights metabolic reprogramming as a central axis governing DCs functionality, with acetyl-coenzyme A (acetyl-CoA) emerging as a pivotal metabolite that integrates lipid synthesis, epigenetic regulation, and mitochondrial energetics to dictate DCs fate in tumors.

Recent advances in single-cell multi-omics and spatial metabolomics have unveiled unprecedented heterogeneity in DCs subsets and their metabolic adaptations. For instance, tumor-infiltrating cDC1s exhibit glycolytic defects under melanoma-associated hypoxia, while cDC2s adopt lipid-laden phenotypes due to SREBP-driven lipogenesis, impairing their cross-presentation capacity [4–6]. Recent studies indicate acetyl-CoA may influences immune cells maturation, migration, and cytokine production by regulating lipid metabolism, oxidative phosphorylation (OXPHOS), and epigenetic modifications [7–10]. This review integrates DCs biology from a metabolic perspective, aiming to offer a mechanistic framework for understanding how metabolic interventions, especially targeting acetyl-CoA, could restore DCs function in cancer therapy. We summarize key research advances based on the following issues: First, while DCs subsets are well—classified, systematic integration of their metabolic remodeling in tumors is lacking. Second, acetyl-CoA's dual roles as an energy source and a chromatin modifier position it as a nexus of DCs functional regulation, yet mechanistic insights remain fragmented. Third, although preclinical metabolic targeting has succeeded, clinical translation lags due to incomplete understanding of TME—specific metabolic crosstalk.

This review begins by delineating the ontogeny and heterogeneity of DCs subtypes to establish the basis for their functional diversity. The next section explores the metabolic regulation of DCs, with a focus on glycolysis, mammalian Target of Rapamycin (mTOR)/AMP-activated protein kinase (AMPK) signaling and lipid metabolism. This is followed by an in-depth discussion of acetyl-CoA's biological roles in DCs. We then proceed to examine DCs dysfunction in various non-tumor diseases, focusing on DCs functional impairment in tumors and the underlying mechanisms. The review emphasizes how acetyl-CoA influences anti-tumor immunity through the regulation of DCs metabolism and function, culminating in therapeutic strategies that exploit DCs metabolic plasticity. This framework links molecular mechanisms to clinical innovation, with a particular emphasis on acetyl-CoA as a key component for developing next-generation DCs therapies.

## Ontogeny and subsets of DCs

DCs are crucial for initiating adaptive immune responses and maintaining immune homeostasis. They originate from pluripotent progenitor cells and differentiate into distinct subpopulations, each with unique functional roles and molecular markers. DCs development is strictly regulated by multiple cytokines. Under the influence of different cytokines, DCs precursors are induced to differentiate into distinct subpopulations. This may be due to the activation of different signaling pathways and metabolic changes. Environmental stimuli, such as those in the tumor microenvironment, can also regulate DCs differentiation, leading to functional heterogeneity [11]. Therefore, understanding the individual development and subpopulation characteristics of DCs is crucial for developing effective immunotherapies [12].

### Developmental pathways

DCs are typically categorized into four functionally distinct subsets: cDC1, cDC2, pDC, and moDC. While their direct precursor cells differ, all DCs subsets originate from multipotent progenitors [13]. The cytokine FMS-like tyrosine kinase 3 (FLT3) plays a pivotal role in determining DCs fate during development. Studies in humans and mice confirm that FLT3 drives hematopoietic progenitor cell differentiation into DCs and sustains DCs homeostasis both in vitro and in vivo [14, 15]. Granulocyte–macrophage colony-stimulating factor (GM-CSF), another critical regulator, strongly promotes moDCs induction in vitro [16, 17]. Unlike GM-CSF, FLT3-mediated DCs differentiation does not favor specific subsets but simultaneously generates multiple cDCs and pDCs populations. Combined stimulation with GM-CSF and FLT3L in vitro preferentially directs cell differentiation toward cDCs subsets [18] (Table 1 [19–25]).

**Table 1:** Characteristics and functions of different DCs subsets

DCs subsets		Location	Makers (Human)	Function	Production	Chemokines
**cDC1**		Lymph, peripheral tissues, blood	CD11c, MHC-II, BDCA3, XCR1, CADM1, Clec9A, BTLA, TLR8, TLR10, IDO2, IDO1, FLT3, CLNK, CD26, CD205	Cross-expression of exogenous antigens in MHCI Immunity of Th1 and CD8^+^ T cells to intracellular pathogens and tumors	INF-λ, IL-27, IL-12, INF-γ, CXCL9, IL-6, IL-10, TNF-α, IL-1β, CXCL10	CCL5, XCL1, CCL7
**cDC2**	**DC2**	Lymph, peripheral tissues, blood	CD11c, HLA-DR, CD1c, SIRPA, CD11b, TLR1, TLR2, TLR3, TLR7, CLEC10A, FceR1A	Direct expression of exogenous antigens on MHCII Activation of immunogenic CD4^+^ Th and regulatory T cells to clear extracellular pathogens	IL-12, INF-γ, IL-18, TNF-α, IL-6	CCL21, CCR7
	**DC3**	Lymph, peripheral tissues, blood	CD36, CD163	Significant activity in polarizing CD8^+^ T cells to CD8^+^CD103^+^ tissue-resident T cells	S100A8, S100A9, INF-γ, IL-12	CCL21, CCR7
**CD16**^**+**^**DC**		Blood	CD16, CD85d, CD85h, CD115, CD31, SLC7A7	Uptake processing, antigen presentation, immune tolerance	Siglic-10, TNF, IL-10	No reported
**pDC**		Lymphoid Residue, blood, tonsils	BDCA2, BDCA4, CLEC4C, BTLA, ILT7, CCR9, SCA1, PTPRS	Type I interferon secretion, antiviral response	IFN-I, IL-3, IL-6, TNF-α	MIP-1α, CXCL9, MIP-β
**LC**		Epidermis, lymph nodes	Langerin, CD1a, E-Cadherin, EPCAM, CD205, HLA-DR,	Involved in non-specific immune, acquire exogenous antigens	TNF, IL-12, CCR6	CCL20
**moDC**		Derive from monocytes	CD11c, HLA-DR, CD14, MMR, CD11b, CD64, CD1c, CD1a	Environment-dependent activation of CD8^+^ T cells, Th1, Th2 and Th17-type immunity	TNF-α, IL-23, NO, MCP-1, CXCL10	CCL2, CCL3

Specific subsets can be distinguished by their unique transcription factor profiles. cDCs subsets originate from pre-cDCs differentiation downstream of common DCs progenitors, a process governed by transcription factors such as interferon regulatory factor (IRF) 8, BATF3, and ID2 [26]. In the cDC2s subset, IRF4 and ZEB2 emerge as critical regulators of differentiation [27]. pDCs develop from pre-pDCs through mechanisms involving E proteins, RUNX1, and IRF8, while also expressing genes typically found in B cells [13, 28]. moDCs represent an inflammatory DCs subset sharing markers with cDC2s and deriving from monocytes downstream of MDP [29]. Single-cell sequencing has revealed DCs markers and developmental pathways across different contexts, including the discovery of mregDCs—a TME-enriched subset expressing immune regulatory molecules—and the delineation of DC2s and DC3s subsets that clarify DCs functional diversity [30, 31].

Environmental stimuli drive DCs differentiation into distinct phenotypes, generating functional heterogeneity among subsets. Tumor-derived prostaglandin E2 (PGE2) suppresses DCs progenitor development, while (transforming growth factor) TGF-β and IL-10 promote tolerogenic cDCs phenotypes [32–34]. By impairing DCs function, tumors disrupt immune response initiation and facilitate immune escape. These findings underscore how environmental modulation during DCs development can alter differentiation trajectories and functional outcomes—a key consideration for improving tumor-associated DCs (TADCs) efficacy.

### Subset characteristics

Distinct subsets of DCs express specific surface molecules that enable their identification and reflect their specialized functions. Human cDC1s are typically identified by the markers CD11c (low), HLA-DR^+^, Clec9A^+^, XCR1^+^, and CD141^+^ [35, 36]. Despite their relatively low abundance among DCs subsets, cDC1s serve as critical antigen-presenting cells in antitumor immunity by capturing damage-associated molecular patterns (DAMPs) from primary tumors. These cells process tumor antigens through HLA-I molecules, migrate to lymph nodes, and present antigens to CD8^+^ T cells to initiate tumor-specific cellular immunity [11, 36]. cDC1s also secrete tumor necrosis factor (TNF)-α, IL-6, IL-12, and type I interferons (IFN-I) to amplify immune cell activation [37–39], while their production of CXCL9 and CXCL10 recruits T cells to tumor sites [40]. The STING signaling pathway is functionally linked to cDC1s activity, with the DCs-STING-IFN axis contributing significantly to various therapeutic outcomes [41–43]. Unlike other DCs subgroups, cDC1s uniquely express Toll-like receptor (TLR)1/3/6/8/10, conferring distinct molecular pattern recognition capabilities for detecting viral and intracellular pathogens [35, 44, 45]. cDC2s primarily activate CD4^+^ T cells via HLA-II, subsequently enhancing CD8^+^ T cell responses and cytotoxic consolidation [46, 47], with human cDC2s identified by CD11c^+^, CD11b^+^, HLA-DR^+^, and CD1c^+^ markers [26, 35]. Upon activation, these cells release IL-12 and IL-1β to stimulate Th17, Th1, and Th2 responses, modulating diverse immune functions [48, 49]. Evidence suggests cDC2s contribute to tumor vaccine efficacy, ICI responses, and immune memory maintenance [47, 50, 51], with TLR2 expression governing their pathogen recognition in anti-infection immunity [35].

Human pDCs are characterized by HLA-DR^+^, CD123^+^, CD303^+^, CD304^+^, and CCR2^+^ markers and specialize in antiviral immunity through IFN-I production [26, 36]. Surface TLR7 and TLR9 activation triggers mTOR and NF-κB pathways upon nucleic acid recognition, inducing IFN-I secretion [25, 52, 53]. While IFN-I can promote antitumor immunity, pDCs correlate with poor prognosis in melanoma and breast cancer [54–56]. Tumor-associated pDCs stimulated by TGF-β express indoleamine 2,3-dioxygenase (IDO) to induce tolerance and employ ICOSL-mediated Treg differentiation to suppress immunity [57, 58]. However, TLR-activated pDCs can regain antigen-presenting capacity and stimulate antitumor responses [59].

**Fig. 1:**
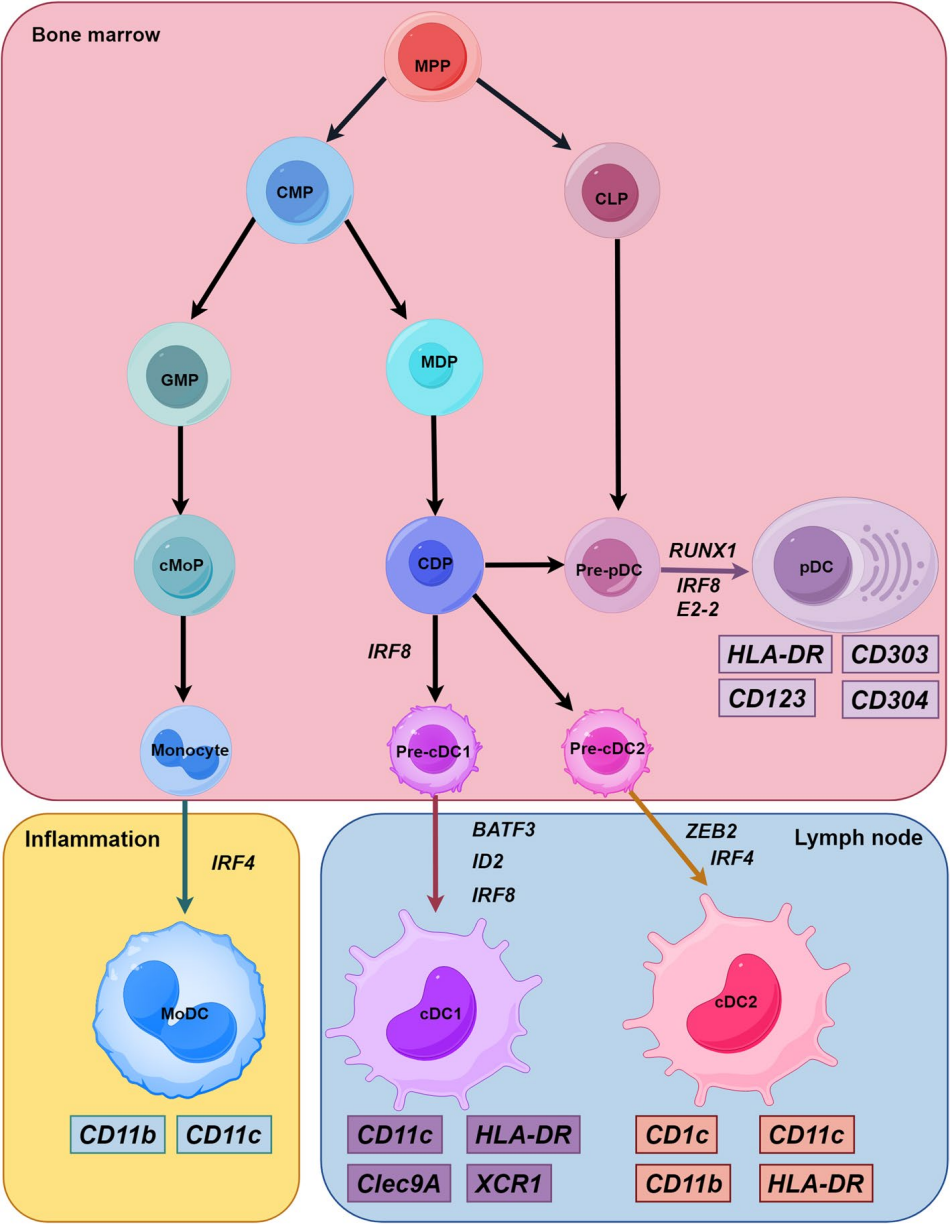
Differentiation and development of DCs. MMP differentiates into CMP and CLP in the bone marrow. CMP differentiates into GMP and MDP, with GMP differentiating into monocytes and becoming moDC in inflammation. MDP differentiates into CDP, which differentiates into pre-pDC and pre-cDC. Pre-cDC is the precursor of both cDC1 and cDC2. It differentiates towards cDC1 under the control of IRF8 and towards cDC2 under the control of IRF4. The differentiation of pre-pDC into pDC depends on transcription factors like E2-2. DC subsets express markers and perform functions. CDP, common DC progenitors; CLP, common lymphoid precursor; CMP, common-myeloid progenitors; MDP, Monocyte-dendritic cell progenitors; MPP, multipotent blood progenitors

moDCs and cDC2s share common surface markers such as CD11c, CD11b, and CD1c, with moDCs differentiating from monocytes under inflammatory conditions [29]. moDCs promote inflammation by inducing IL-17 secretion from CD4^+^ T cells, which drives Th17 cell differentiation [60]. These cells also initiate Th2-mediated immune responses that contribute to allergen-induced inflammation [61]. Within tumors, moDCs infiltration enhances cytotoxic lymphocyte (CTL) activity through nitric oxide synthase (iNOS)-mediated nitric oxide production, facilitating tumor cell killing [62, 63]. moDCs further demonstrate the capacity to activate T cells and improve ICI responses [64–66]. In the TME, moDCs enable antigen cross-presentation and subsequent T-cell activation, a mechanism exploited by DCs-based vaccines for cancer therapy [67, 68]. Immune checkpoint blockade in melanoma models revealed that tumor-infiltrating lymphocyte efficacy strongly correlated with moDCs populations expressing elevated CD80, CD86, and MHC-II. These moDCs supported tumor-infiltrating lymphocyte expansion via iNOS expression, suggesting monocyte-to-moDCs differentiation could potentiate antitumor immunity [68–70] (Fig. 1).

## Metabolic regulation of DCs

The metabolic regulation of DCs has been extensively studied, revealing inherent variations among DCs subsets. cDC1s and moDCs demonstrate stronger mitochondrial function and OXPHOS capacity compared to cDC2s and pDCs, potentially explaining their divergent responses to identical stimuli in both physiological and experimental settings [71–73]. Metabolic profiles further differ across DCs developmental stages: early activation involves concurrent glycolysis and fatty acid oxidation (FAO)-dependent OXPHOS elevation, while prolonged activation shifts metabolism toward exclusive glycolytic dependence as OXPHOS capacity diminishes [74–76]. This metabolic transition coincides with phenotypic maturation, characterized by enhanced cytokine secretion (including IL-6 and IL-12) and upregulated CD80/CD86 expression [77]. Current evidence suggests iNOS-derived NO suppresses OXPHOS by inhibiting the electron transport chain [78, 79], while GM-CSF and FLT3L stimulation activates the mTOR-hypoxia inducible factor (HIF)−1α axis to promote glycolytic enzyme activity [80–82]. Understanding how DCs metabolism shapes functional outcomes across biological contexts remains essential for identifying novel therapeutic targets.

### Metabolic pathways

#### Glycolysis

Glycolysis plays a critical role in DCs maturation by supplying both ATP and essential metabolic intermediates that maintain DCs functionality. The glycolysis inhibitor 2-deoxyglucose reduces CD80/CD86 co-stimulatory molecule expression, suppresses TNF-α and IL-12 secretion, and impairs DCs migratory capacity in both ex vivo and in vivo models through downregulation of CCL7 [77, 83], ultimately compromising T cell activation. Glycolytic metabolites further contribute to DCs maturation, with mitochondrial pyruvate import representing a particularly crucial step in this process [75, 77]. Citrate derived from pyruvate via the tricarboxylic acid (TCA) cycle generates cytoplasmic acetyl-CoA for lipid biosynthesis [75], which supports DCs physiological functions by maintaining ER and Golgi apparatus activity for proper cytokine secretion and antigen processing [75, 77, 84]. Under LPS stimulation or IL-4 activation, DCs primarily utilize glycogen reserves to fuel glycolysis before upregulating glucose transporter protein1 for external glucose uptake [85, 86]. This imported glucose undergoes glycogen synthesis followed by glycolytic breakdown, completing the metabolic reprogramming characteristic of DCs activation [83, 85]. Early LPS-induced glycolytic activation depends on TBK1/IKKε/AKT-mediated hexokinase II stimulation [77], while sustained enzyme expression (including phosphofructokinase and glucose transporter protein1) is regulated by PI3K/AKT/mTOR pathway activation and AMPK pathway inhibition [74, 83].

#### mTOR and AMPK

The influence of mTOR on DCs depends on their developmental stage. mTOR pathway activation during DCs differentiation and maturation supports their proper development. Rapamycin-mediated mTOR inhibition in vitro decreases the generation of cDC1s, cDC2s, and pDCs subsets [80, 81], yet paradoxically enhances the functional capacity and T-cell stimulatory potential of mature bone marrow-derived DCs in mice. Genetic ablation of TSC1 in DCs disrupts MHC-I and IL-7 expression through mTOR hyperactivation, compromising T-cell homeostasis [87–89]. These divergent outcomes likely reflect distinct metabolic requirements at different DCs life stages. The AMPK pathway counteracts mTOR signaling [90], with AMPK activation in tumor-associated DCs typically inducing tolerogenic properties characterized by improved mitochondrial function, increased FAO and OXPHOS, and diminished CD80/CD86 expression [3, 91, 92]. Exogenous AMP additionally stimulates DCs A2b receptors, triggering TGF-β and IL-10 production – immunosuppressive cytokines that may exert their inhibitory effects through AMPK pathway engagement [3, 93–97]. Conversely, extracellular ATP drives DCs inflammatory polarization by promoting IL-1β secretion for T-cell priming while limiting adenosine accumulation and ameliorating hypoxic stress [98–100].

#### Lipid metabolism

LPS-activated lipid accumulation in DCs correlates with enhanced antigen cross-presentation, a pivotal mechanism for initiating tumor-specific immune responses [101]. While lipid accumulation in DCs generally supports T cell activation, excessive lipid buildup in TADCs disrupts their normal functions [102]. The origin of these lipids remains unclear, though potential sources include external lipid uptake via macrophage scavenger receptors (Msr) or endogenous synthesis driven by tumor-induced metabolic reprogramming. Melanoma-derived Wnt5a, for instance, activates β-catenin signaling in TADCs, where subsequent PPAR-γ activation enhances OXPHOS coupled with FAO to drive lipid accumulation. Etomoxir-mediated FAO inhibition in DCs has been shown to improve immunotherapy outcomes [8, 103, 104]. These accumulated lipids undergo oxidation upon reactive oxygen species (ROS) exposure, forming oxidized derivatives that impair antigen presentation in murine cDC1s by binding heat shock protein 70 and blocking MHC-peptide complex formation [105]. ROS additionally activates the IRE1α-XBP1 pathway, inducing endoplasmic reticulum (ER) stress that further compromises TADCs-mediated T cell activation while stimulating additional lipid synthesis [104, 106], creating a self-reinforcing cycle of lipid accumulation and oxidative damage.

#### Amino acid metabolism

Tryptophan and arginine metabolism critically regulates DCs functional homeostasis. IDO mediates tryptophan conversion to kynurenine [107], and its expression by TADCs activates Tregs while suppressing immune responses. Kynurenine sustains IDO expression through activation of the DC's aromatic hydrocarbon receptor (AhR) [108, 109]. Treg-DCs interactions via CTLA-4 binding to B7 receptors further enhance IDO expression [110, 111], establishing two positive feedback loops: IDO-kynurenine-AhR-IDO and IDO-Treg-CTLA-4-B7-IDO, both reinforcing immunosuppressive TADCs function. iNOS metabolizes arginine to generate citrulline and NO [112], which modulates cellular behavior within the TME [113, 114]. Polyamines derived from arginase 1-mediated catabolism regulate IDO phosphorylation and signaling in DCs, with TGF-β-stimulated IDO production requiring Arg1 expression [115].

#### Vitamin metabolism

Vitamins regulate essential enzymatic processes and maintain metabolic homeostasis [116], influencing intracellular metabolism and DCs phenotypes through signaling pathway modulation. Vitamin D alters DCs glucose metabolism by increasing PFKFB4 activity, which enhances glucose oxidation [117]. These metabolic shifts promote a tolerogenic DCs phenotype characterized by Treg differentiation while suppressing DCs maturation and inflammatory responses [118]. Vitamin A governs DCs function via retinoic acid metabolism, a pathway critical for maintaining intestinal DCs tolerance and immune homeostasis; dysregulation of this process can intensify intestinal inflammation [119, 120]. As a potent antioxidant, vitamin E protects membrane lipids and modulates ROS/RNS production. Emerging evidence indicates that vitamin E enhances antigen presentation in TADCs through SHP1 targeting [121], potentially amplifying therapeutic responses to chemotherapy and radiotherapy. In obese mouse models, this effect appears mediated through FAO regulation during oxidative stress [122]. Tocopherol-based nanoemulsions facilitate DCs vaccine delivery into the TME by mitigating lipid accumulation and oxidative damage while enhancing T cell recruitment from tumors and lymph nodes [123], demonstrating vitamin E's dual capacity for metabolic and immune modulation.

In summary, metabolic reprogramming drives the functional plasticity of DCs. Glycolysis fuels DCs maturation and migration by generating ATP and metabolic intermediates like pyruvate, with TBK1/IKKε/AKT and PI3K/mTOR signaling cascades regulating key enzymatic steps. mTOR and AMPK pathways display reciprocal regulation during DCs development: mTOR activation stimulates DCs differentiation and pro-inflammatory responses, while AMPK promotes tolerogenic DCs through enhanced FAO and OXPHOS. Lipid metabolism exerts dual effects on DCs function—moderate lipid accumulation improves antigen cross-presentation, whereas TME-induced lipid overload, mediated by β-catenin/PPAR-γ activation, impairs DCs activity via oxidative damage and ER stress. The tryptophan-IDO-aryl hydrocarbon receptor axis and arginine-iNOS/Arg1-polyamine pathway mediate DCs immunosuppression, with IDO-driven regulatory T cell expansion reinforcing immune evasion in tumors. Vitamins influence DCs metabolism and immune responses by modulating enzymatic activity, exemplified by vitamin D's upregulation of PFKFB4, and redox balance through mechanisms like vitamin E's targeting of SHP1. Therapeutic interventions targeting these pathways may counteract DCs dysfunction and inform new approaches to cancer immunotherapy.

### Biological functions of Acetyl-CoA

Acetyl-CoA serves as a central metabolite that fuels the TCA cycle and OXPHOS, enabling complete oxidation of sugars, lipids, and proteins for ATP production [124]. This molecule also acts as a biosynthetic precursor for cholesterol, ketone bodies, and various physiologically active compounds [125]. Within the nucleus, compartmentalized acetyl-CoA levels modulate histone acetylation through lysine acetyltransferase (KAT) activity, a process further influenced by lipid synthesis pathways [126–128]. Recent studies demonstrate that acetyl-CoA exerts broad functional impacts on TME cells through metabolic regulation [7, 129].

#### Source and transport of Acetyl-CoA

Cells synthesize acetyl-CoA through multiple metabolic pathways, such as pyruvate decarboxylation by the pyruvate dehydrogenase complex (PDC), fatty acid β-oxidation, acetate-CoA ligation via acyl-CoA short-chain synthetases (ACSS), branched-chain amino acid degradation, and citrate cleavage by ATP citrate lyase (ACLY) [7, 130–133]. The PDC-mediated conversion of pyruvate remains the primary acetyl-CoA source in most cells, feeding the TCA cycle to sustain OXPHOS and cellular energy production. Ketone body-derived acetyl-CoA not only fuels extrahepatic tissues but also acts as a signaling molecule with potential implications in cancer therapy [7]. Branched-chain amino acid catabolism substantially contributes to acetyl-CoA pools through sequential actions of branched-chain amino acid aminotransferase and branched-chain keto acid dehydrogenase kinase, with these pathways implicated in pancreatic disease pathogenesis [134–136].

Cytosolic acetyl-CoA pools interconnect through derivative intermediates. The citrate-pyruvate cycle mediates acetyl-CoA exchange between mitochondria and cytosol via SLC25A1-mediated citrate transport [137, 138]. Oxaloacetate derived from this cycle undergoes malate conversion before mitochondrial reimport through SLC25A10 and SLC25A11 [139, 140]. Malate-derived pyruvate re-enters mitochondria via the mitochondrial pyruvate carrier [141]. These redundant transport systems maintain compartment-specific acetyl-CoA levels for diverse functions, including nuclear gene regulation under metabolic stress, ensuring cellular homeostasis [7, 142–144].

#### Metabolic pathways and biological roles of Acetyl-CoA

Acetyl-CoA metabolism varies across cell types and plays a critical role in cellular maturation and development. Macrophages and DCs exhibit impaired TCA cycle activity, releasing acetyl-CoA after citrate synthesis for lipid biosynthesis and gene expression regulation—a prerequisite for early cellular activation [76]. In contrast, most cells primarily rely on the intact TCA cycle for energy production. The high-energy thioester bond in acetyl-CoA facilitates acetyl group transfer [145], establishing its role as a key acetyl donor for protein acetylation mediated by enzymes like KAT. Both histone acetylation, which modulates gene expression, and non-histone protein acetylation influence fundamental processes including cell division and autophagy [146–148].

As the initiating substrate for fatty acid synthase (FAS) [149], acetyl-CoA serves essential functions in lipid biosynthesis. Carnitine bridges acetyl-CoA and fatty acid metabolism, with SLC25A20 transporting cytosolic fatty acids as acylcarnitine alongside converted carnitine derivatives, maintaining metabolic equilibrium [128, 150, 151]. These lipids constitute cellular membranes and support organelle expansion in the ER and Golgi apparatus [85, 87], enabling normal physiological functions. Statins, which inhibit cholesterol synthesis, have been shown to impair DCs invasiveness in immune disorders [152–154]. Lipids also function as crucial intracellular signaling molecules [155], with multiple pathways requiring stable lipid levels for proper signal transduction.

Beyond being a glycolytic byproduct, acetyl-CoA orchestrates cellular functionality. Glycolysis-driven epigenetic regulation through acetyl-CoA production allows immune cells to maintain histone acetylation patterns [156–158]. Tumor cells with PTEN-inducible kinase 1 overexpression exhibit suppressed glycolysis and reduced acetyl-CoA levels, leading to apoptotic cell death. Acetyl-CoA further integrates glycolysis with fatty acid metabolism while regulating glycolytic flux [159]. During inflammation, FAO-derived acetyl-CoA enhances glycolysis [160], with acetyl-CoA-mediated acetylation states modulating signaling pathways that drive metabolic reprogramming [161].

Branched-chain amino acid catabolism contributes substantially to acetyl-CoA pools, while TCA cycle intermediates derived from acetyl-CoA serve as precursors for glutamate, arginine, and other amino acids [162]. Fluctuations in acetyl-CoA and amino acid levels reflect cellular nutrient status, enabling metabolic regulation through diverse signaling pathways [162, 163].

**Fig. 2:**
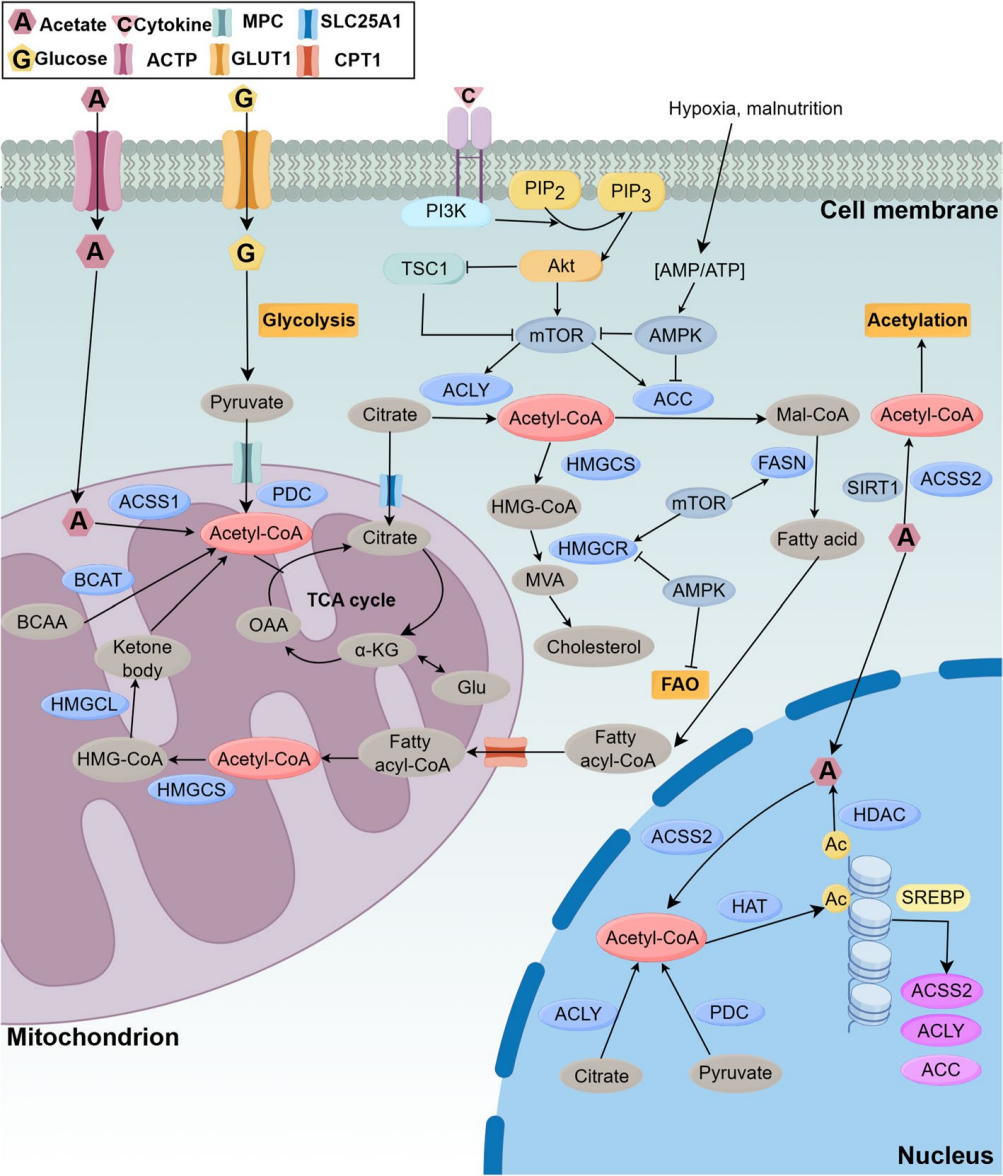
Acetyl-CoA related metabolism. Glucose produces pyruvate, which is converted into acetyl-CoA and enters the TCA cycle, providing the conditions for OXPHOS to occur. In mitochondria, acetic acid and BCAA serve as additional sources of acetyl-CoA. The intermediate metabolites generated by acetyl-CoA in the TCA cycle exert a significant influence on cellular metabolism and function. α-KG can be interconverted with Glu, and citrate can be used as an acetyl-CoA carrier to enter the cytoplasm and participate in lipid metabolism. Citric acid is broken down into acetyl-CoA, which is used to produce fatty acids and cholesterol. Fatty acids can be converted to acetyl -CoA through FAO. Additionally, acetyl-CoA have various source within the nucleus. Acetyl-CoA provides acetyl groups for histone acetylation and regulates gene expression. Furthermore, the expression and activity of acetyl-CoA related enzymes are regulated by transcription factors and signaling pathways. ACTP, acetate transporter protein; α-KG, α-ketoglutaric acid; BACA, branched chain amino acid; BCAT, branched-chain amino acid aminotransferase; CPT, Carnitine acyl transferase; HMGCL, HMG-CoA lyase; HMGCS, HMG-CoA synthase; MVA, Mevalonate; OAA, oxaloacetic acid

Acetyl-CoA both depends on and regulates enzymatic activity. Declining acetyl-CoA concentrations reduce the activity of acetyl-CoA carboxylase (ACC) isoforms ACACA and ACACB, along with HMG-CoA reductase [164, 165]. The acetyl-CoA/CoA ratio additionally modulates KAT enzymes, including KAT2A (GCN5), KAT2B (PCAF), and p300/CBP—key regulators of both metabolism and immune function [166, 167]. These findings establish acetyl-CoA as a central integrator of carbohydrate, lipid, and amino acid metabolism, coordinating metabolic states through acetylation processes and mTOR pathway modulation to maintain cellular functional homeostasis (Fig. 2).

### Role of Acetyl-CoA in DCs development and differentiation

The regulatory role of metabolic pathways in DCs biology has been well documented, with acetyl-CoA emerging as a key metabolic regulator due to its broad influence on cellular metabolism. During in vitro DCs differentiation induced by GM-CSF and FLT3L, metabolic reprogramming occurs through modulation of the mTOR and AMPK signaling pathways, which respectively promote anabolic and catabolic states [91, 168, 169]. Acetyl-CoA deprivation triggers AMPK activation while suppressing mTOR signaling [170], suggesting this direct pathway mediates acetyl-CoA's effects on DCs function. Genetic studies demonstrate that TSC1 deficiency activates mTOR/PPAR-γ signaling, enhancing neuropilin-1 expression and naïve T-cell proliferation while impairing antigen presentation and specific T-cell activation. TSC1 additionally regulates MHC-II expression through IRF4 and CIITA [171–173]. The TSC1/mTOR axis not only controls acetyl-CoA levels by modulating metabolic enzyme activity but also determines its functional utilization, positioning acetyl-CoA as a critical downstream effector in this pathway [170, 174].

In co-cultures of GM-CSF-derived macrophages and DCs, macrophage-derived NO inhibits OXPHOS while promoting DCs glycolysis. While moderate NO production sustains glycolytic metabolism during DCs activation, excessive NO impairs acetyl-CoA-dependent histone acetylation [175–177], indicating NO may mediate acetyl-CoA's regulatory effects. Activated DCs accumulate lactate, which is exported via monocarboxylate transporters [3, 75], creating an acidic microenvironment that attenuates DCs-mediated T-cell activation [178]. Intracellular acidification similarly enhances histone deacetylase activity [179], potentially reducing acetyl-CoA-dependent acetylation of immune genes and compromising DCs function.

DCs respond to acetyl-CoA-derived metabolites including citrate, fatty acids, and succinate by altering their functional properties. Succinate receptor signaling enhances DCs migration and TNF-α secretion [76, 180–182], while lauric acid upregulates CD80/CD86 expression [183]. In contrast, n-3 polyunsaturated fatty acids and palmitic acid inhibit DCs maturation and induce an IL-23-driven transcriptome that promotes Treg-mediated immunosuppression [183–185].

**Fig. 3:**
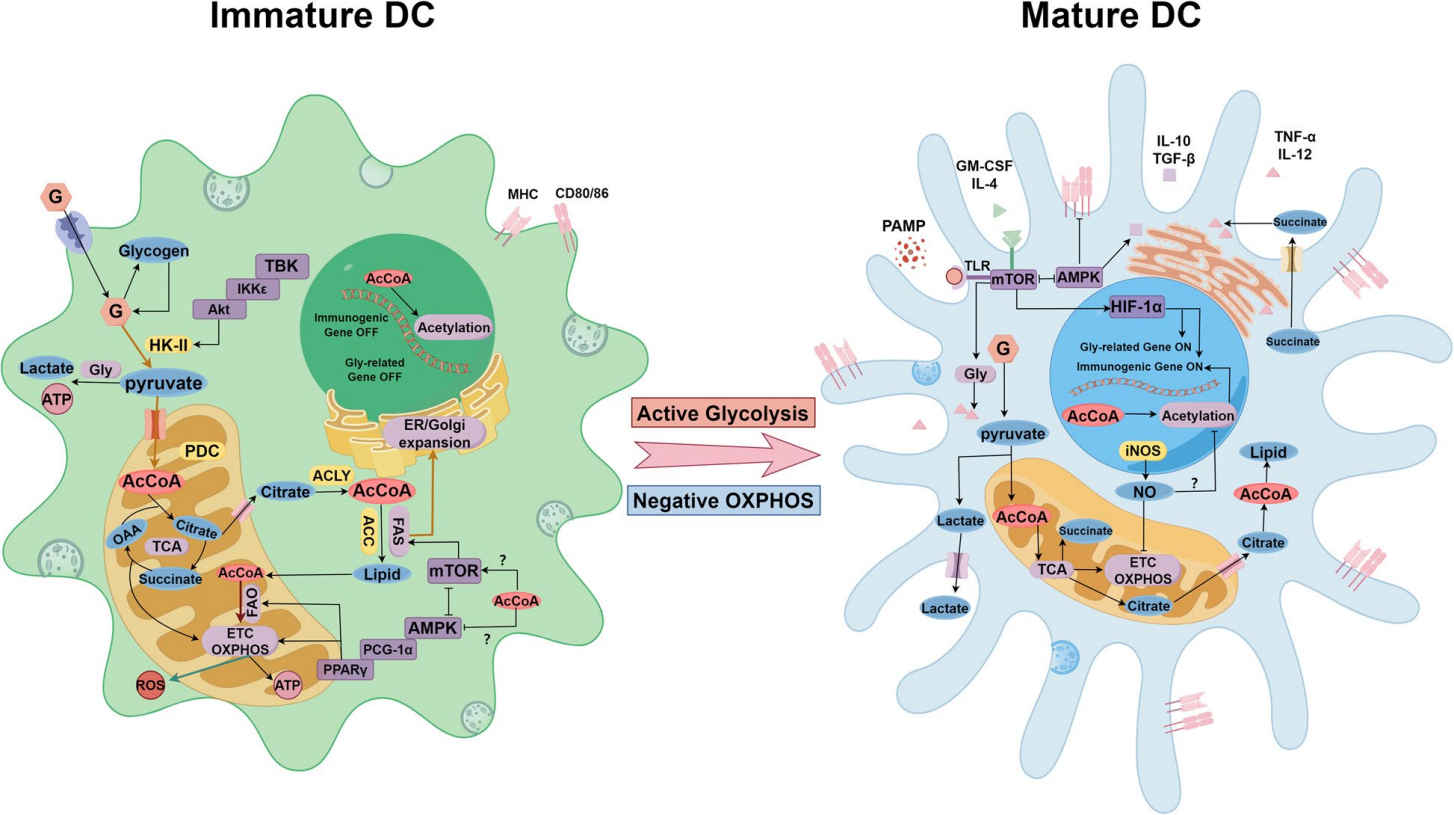
Metabolic differences between different DC states and the regulatory role of acetyl-CoA. As DCs mature, their metabolic profile shifts. Immature DCs initially rely on glycolysis, driven by hexokinase II activated via the TBK-IKKε pathway. Pyruvate, generated from glucose, is converted to acetyl-CoA by PDC and enters the TCA cycle. The electron transport chain and OXPHOS generate ATP to support early DC differentiation, particularly into cDC1. During activation, enhanced OXPHOS and ROS production are crucial for cDC1 and cDC2 differentiation, respectively. Citrate from the TCA cycle is transported to the cytoplasm and converted to acetyl-CoA by ACLY, then to fatty acids by ACC, aiding ER and Golgi expansion. Upon sufficient antigen activation and cytokine stimulation, mature DCs maintain high glycolysis levels, supporting cytokine secretion and migration to lymph nodes. This is regulated by mTOR pathway activation, driving gene expression changes via HIF-1α. HIF-1α and iNOS-derived NO inhibit OXPHOS in mature DCs, while AMPK counteracts the effects of mTOR. The effect of acetyl-CoA on overall metabolic status may regulate mTOR and AMPK. Mature DCs exhibit elevated cytokine secretion and T cell activation but reduced antigen uptake and phagocytosis. Acetyl-CoA derivatives also regulate DC surface molecule expression and cytokine secretion

These findings collectively demonstrate that acetyl-CoA influences DCs biology through both direct signaling pathway activation and indirect metabolite-mediated mechanisms. The diverse functional outcomes depending on specific metabolites underscore the complexity of acetyl-CoA's role in DCs maturation and differentiation, highlighting the need for precise targeting in therapeutic applications (Fig. 3).

## DCs in non-oncological diseases

It is evident that DCs fulfil a pivotal function in preserving immune system homeostasis and modulating immune responses. They play a pivotal regulatory role in immune activation and tolerance through antigen presentation and cytokine secretion. In recent years, numerous studies have revealed the diversity and complexity of DCs in autoimmune diseases, allergic diseases, transplant immunology, infectious diseases, and chronic inflammatory diseases. This section provides a comprehensive overview of the functional characteristics, immune regulatory mechanisms, and associations with disease progression of DCs in different disease contexts. In addition, it explores their potential applications in disease treatment.

### Autoimmune disease

DCs serve as pivotal regulators of both immune activation and tolerance, maintaining systemic homeostasis. In the thymus, DCs promote central tolerance by presenting self-antigens via MHC-II, enabling the clearance of autoreactive CD4^+^ T cells [186]. Peripheral DCs further enforce tolerance by eliminating self-reactive CD8^+^ T cells through clonal deletion, incompetence, or Treg induction [187]. Breakdown of these mechanisms underlies the immune dysregulation characteristic of autoimmune diseases. Rheumatoid arthritis (RA) synovium harbors inflammatory DCs that drive pathogenic Th1/Th17 differentiation, a hallmark of disease progression [188]. Both RA and systemic lupus erythematosus (SLE) exhibit DCs accumulation at inflammatory sites, mediated by CCL20-dependent recruitment [189–192]. SLE patients demonstrate particularly pronounced pDCs infiltration in affected skin and kidneys, implicating these cells in tissue-specific pathology [193, 194]. Dermatomyositis lesions show CCR7-deficient DCs that fail to migrate to lymph nodes, promoting their pathological retention and disease exacerbation [195]. Within RA synovium, DCs-derived IL-23 and IL-12 sustain Th1/Th17 dominance over regulatory subsets, perpetuating tissue damage [196]. IL-17 emerges as a central mediator of cartilage destruction, both by amplifying inflammation and directly inducing matrix-degrading enzymes [197–199]. Hypoxia in synovial tissue disrupts OXPHOS and FAO while augmenting glycolysis in cDCs, leading to increased immunoreactivity and enhanced T-cell activation. Conversely, the unique metabolic characteristics of pDCs, likely explain their minimal role in driving inflammation in RA [200]. Metabolite-driven DCs polarization occurs in RA, where succinate sensing triggers pro-inflammatory pathways [201]. In SLE, pDCs initiate autoimmunity via IFN-I-induced B-cell activation and autoantibody production [202]. Metabolic disturbances involving mTOR, ROS, and mitochondrial dysfunction further impair pDCs regulation, with oxidized mtDNA promoting aberrant IFN responses that amplify autoreactive lymphocyte activity [203].

### Allergic diseases

DCs are pivotal in allergic responses, orchestrating allergen interactions and T-cell activation. PPAR-γ serves as a critical regulator of DCs-mediated allergic inflammation, with pharmacological activation attenuating DCs responsiveness to respiratory allergens and mitigating Th2-driven airway inflammation [204]. This mechanism involves PPAR-γ-mediated suppression of NF-κB signaling, which diminishes IL-12 production and curbs Th2 differentiation [205]. Elevated FAO is activated by PPAR-γ, fostering tolerogenic DCs development [206]. Pulmonary conventional DCs primarily engulf and process allergens during hypersensitivity reactions before migrating to lymph nodes to prime antigen-specific T-cell responses. Conversely, plasmacytoid DCs promote fungal spore clearance and restrain allergic responses via Treg activation and IL-10 secretion [207, 208]. Langerhans cells, the epidermal DCs population, are fundamentally implicated in atopic dermatitis pathogenesis. Alongside dermal DCs, they drive disease progression by recruiting Th cells through CCL17 and CCL22 chemokine production [209]. DCs-initiated Th2 immunity constitutes a central pathway in atopic dermatitis development, propelled by inflammatory cytokines (IL-31, IL-33, IL-22, IL-4) that promote T-cell recruitment and activation via DCs crosstalk [210]. DCs additionally amplify Th2 and Th22 expansion through non-integrin molecule uptake and DCs-SIGN-mediated IL-6 and TNF-α release [211]. Th subset distribution evolves dynamically during atopic dermatitis progression: Th2 cells dominate acute-phase inflammation, while chronic lesions exhibit mixed Th1, Th17, and Th22 infiltration [212].

### Transplantation immunity

In organ transplantation, DCs exhibit dual regulatory roles in allograft rejection through both immunosuppressive and immunostimulatory mechanisms. Hepatic DCs typically demonstrate a tolerogenic phenotype that suppresses immune responses [213], yet during liver transplantation, they shift to a pro-inflammatory state upon exposure to mediators such as TNF-α, IL-6, and activated pattern-recognition receptors. These inflammatory signals correlate strongly with ischemia–reperfusion injury common in transplantation procedures [214, 215]. Mature DCs elevate expression of co-stimulatory molecules, chemokines, and adhesion molecules, enhancing alloantigen presentation and antigen-specific T-cell cytotoxicity. Hepatic cDCs transfer into alloantigen-specific recipients under steady-state conditions induces IL-10-mediated immune tolerance [216]. In kidney transplant rejection, donor and recipient DCs serve distinct functions: recipient DCs present alloantigens while donor DCs facilitate antigen transport. MHC molecule transfer between donor and recipient DCs enables recipient T-cell activation and subsequent graft injury [217]. Ischemia–reperfusion injury activates donor DCs to drive acute rejection, whereas recipient DCs predominantly mediate chronic rejection [218, 219]. Renal cDC2s promotes chronic injury through antibody-dependent pathways involving B-cell activation and antibody production in transplant contexts [220].

### Infectious disease

#### Infections caused by bacteria

Helicobacter pylori secrete ADP-heptose, a metabolic product that attenuates IFN-I signaling during bacterial infection, disrupting DCs activation feedback and impairing CD8^+^ T cell responses to facilitate chronic infection through TLR2-dependent mechanisms [221]. This DCs dysfunction compromises antitumor immunity, as evidenced by reduced tumor-specific T cell priming in infected mice and shorter progression-free survival among H. pylori-positive lung cancer patients receiving PD-1 blockade therapy [222]. The pathogen simultaneously drives gastric inflammation via its virulence factor CagA, which triggers NF-κB activation in DCs, elevating IL-6 production and promoting IL-17A^+^ T cell differentiation [223]. These findings reveal how H. pylori balances immune evasion with inflammatory responses through DCs modulation. Therapeutic strategies targeting bacterial metabolites to restore DCs function or modifying microbiota composition may overcome immunosuppression and improve treatment outcomes.

In tuberculosis, DCs play a pivotal role in Mycobacterium tuberculosis (MTB) antigen presentation. Following granuloma formation, DCs transfer antigens to peripheral counterparts, maintaining T-cell activation in draining lymph nodes [224, 225]. TB patients exhibit defective DCs-mediated T-cell priming, resulting in diminished pulmonary T-cell recruitment and markedly lower circulating DCs counts than healthy individuals [226–228]. MTB further subverts host defenses by altering DCs differentiation, increasing moDCs while decreasing IFN-γ-producing CD4^+^ T cells critical for bacterial clearance [229]. These moDCs display an immunosuppressive profile characterized by reduced IL-12 and elevated IL-10 secretion [230]. Paradoxically, pDCs in TB patients produce abundant IFN-I while adopting anti-inflammatory lung phenotypes, potentially suppressing protective immunity through IL-12 downregulation—a hypothesis supported by post-treatment normalization of pDCs numbers and IL-12^+^ DCs frequencies [231–233]. Active TB additionally features DCs with upregulated BTLA expression, which correlates with diminished CD80, CD83, and IL-12 production, impairing antigen presentation. These BTLA^+^ DCs further promote immune tolerance via TGF-β-dependent regulatory T cell induction [234].

#### Infections caused by viruses

HIV infection causes AIDS by severely compromising the immune system, with CD4^+^ T cells serving as the primary viral target. DCs play a pivotal role in HIV pathogenesis, frequently encountering the virus during early infection [235]. These cells recognize HIV through both CD4 and DCs-SIGN receptors [236–238]. Following viral uptake, DCs present HIV antigens to CD4^+^ T cells via MHC-II, primarily through DCs-SIGN interactions with the gp120 glycoprotein [235, 237]. The virological synapse conversely facilitates HIV transfer from DCs to CD4^+^ T cells, mediated by CD4 and CCR5 binding to gp160 [239, 240]. The antigen presentation capacity of DCs may relate to HIV's N-glycan composition, with increased mannose content potentially enhancing this function [241, 242]. Gp120-exposed plasmacytoid DCs exhibit impaired TLR9 signaling. While viral stimuli normally promote DCs maturation through inflammatory pathways, HIV suppresses TLR-mediated activation in DCs, as demonstrated in multiple studies. TLR agonists could therefore mitigate HIV's suppressive effects by improving viral internalization and immune response initiation in DCs [243, 244].

In HCV infection, DCs provide critical immune defense by detecting viral RNA through TLR7, stimulating IFN and interleukin production to modulate immunity [245]. pDCs recognize RNA from infected hepatocytes, inducing IFN-I production to amplify immune responses [238]. HCV infection diminishes peripheral plasmacytoid DCs counts and disrupts DCs functionality. The virus modifies co-stimulatory molecule and MHC-II expression, modulates IDO via the JAK-STAT pathway, and elevates inhibitory molecules (ICOS, PD-L1), collectively suppressing T-cell activation [246–248]. Studies demonstrate that HCV Core and NS proteins impair DCs function through TLR2 activation, reducing co-stimulatory molecule expression, compromising antigen presentation, and diminishing IL-12 secretion, thereby weakening DCs-mediated immune activation [245, 249].

#### Infections caused by fungal pathogens

Fungal infections persistently colonize immunocompromised hosts, contributing to substantial mortality and representing a major global health burden [250]. In atopic dermatitis, Malassezia promotes DCs maturation through macrophage mannose receptor-mediated uptake, eliciting a Th2-biased immune response characterized by IL-1β and TNF-α secretion with limited IL-12p70 production. This pathogen further alters DCs-NK cell interactions by suppressing NK cell cytotoxicity while inhibiting DCs apoptosis, thereby perpetuating chronic inflammation [251]. Emerging evidence demonstrates that Malassezia engages DCs through C-type lectin receptors, triggering SYK/CARD9-dependent potassium efflux that activates the NLRP3 inflammasome and IL-1β release, ultimately polarizing Th17/Th22 responses [252]. Lipid antigens derived from both follicular CD1a^+^ Langerhans cells and Malassezia's Mal f 1 allergen stimulate DCs activation via C-type lectin receptors-1, enhancing expression of TNF-α, IL-1β, and co-stimulatory markers including CD80, which may sustain Th22/Th17-driven inflammation [253, 254].

The opportunistic pathogen Candida induces divergent DCs responses depending on its morphological state: yeast forms stimulate Th1-polarizing IL-12 production, whereas hyphae suppress IL-12 while inducing Th2-associated IL-4, demonstrating morphology-dependent immunomodulation [251]. Candida albicans additionally subverts DCs metabolic pathways by modulating farnesol secretion, which impairs mitochondrial respiration during moDCs differentiation, accelerates lipid droplet accumulation, and compromises protective T cell priming [255]. Future investigations should delineate fungus-specific signaling mechanisms and develop targeted strategies to disrupt these pathogenic pathways.

#### Infections caused by parasites

DCs serve as critical orchestrators of adaptive immunity in parasitic infections, demonstrating distinct functional adaptations depending on the protozoan or helminth context. We examine DCs-parasite dynamics using leishmaniasis and helminthiasis as model systems. During Leishmania infection, DCs detect pathogen-associated molecular patterns such as lipophosphoglycan through TLR2/4 receptors, triggering p38/JNK MAPK pathways that promote Th1 polarization via IL-12 production [256–258]. Certain species like L. amazonensis disrupt this process by preferentially activating ERK1/2 signaling over p38/JNK, thereby suppressing IL-12 secretion and sustaining pathogenic Th2 responses [259]. The parasite further manipulates host immunity through TLR2-mediated induction of SOCS1/3, which inhibits JAK/STAT signaling and subsequent proinflammatory cytokine release [260]. Interestingly, Leishmania-secreted extracellular vesicles demonstrate both immunostimulatory and suppressive effects, simultaneously enhancing moDCs activation and CD8^+^ T cell recruitment while facilitating immunosuppressive DCs-NK cell communication [261, 262].

In helminth infections, Schistosoma egg antigens stimulate DCs-SIGN/TLR4 to preferentially activate ERK1/2 rather than p38 MAPK, shifting cytokine production toward IL-10 at the expense of IL-12 [263, 264]. Helminth-derived factors modulate DCs surface markers, upregulating OX40L and PD-L2 to enhance Th2 priming through PD-1 engagement. Ascaris cystatin exemplifies this immunomodulation by simultaneously promoting SREBP-dependent cholesterol biosynthesis and inhibiting CD80 expression [265], and while chronic hookworm infection expands plasmacytoid DCs populations to sustain systemic immune tolerance [266]. Understanding the precise spatiotemporal regulation of DCs-helminth interactions may reveal novel approaches to restore immune homeostasis, potentially leveraging parasite-derived molecules for therapeutic immunomodulation without compromising protective immunity.

### Chronic inflammatory diseases

DCs critically influence chronic inflammatory diseases, particularly in establishing intestinal immune tolerance. Their dysfunction strongly correlates with inflammatory bowel disease (IBD) pathogenesis, encompassing both ulcerative colitis (UC) and Crohn's disease (CD). pDCs accumulate at lesion sites and contribute to IBD initiation. CD patients exhibit increased mucosal pDCs infiltration and elevated IL-1β concentrations. Peripheral blood pDCs from IBD patients demonstrate higher CD40/CD86 ratios along with enhanced secretion of IL-6, IL-8, and TNF-α [267, 268]. TNF-α not only drives IBD progression but also suppresses IL-22 production in DCs, impairing mucosal repair. The reduction in DCs populations during acute IBD phases implies their depletion occurs before mucosal damage becomes evident [269–271]. CD103^+^ DCs in both CD and UC patients show upregulated PD-L1 expression, increased pro-inflammatory cytokine release, and enhanced T-cell activation, likely resulting from Smad7 deficiency and impaired TGF-β signaling [272]. NAFLD represents a major pathway to liver fibrosis and cirrhosis, with its progression involving dysregulated innate immunity through T cells, neutrophils, macrophages, and DCs [273]. Heightened cDC1s activity in non-alcoholic steatohepatitis parallels disease severity and promotes progression by triggering metabolic reprogramming in inflammatory T cells within hepatic lymph nodes [274]. The contribution of DCs to NAFLD pathogenesis remains debated across experimental models. Methionine-choline deficient diet models implicate DCs in pro-inflammatory responses, while contrasting evidence suggests they may exert protective effects [275–277]. Emerging data indicate DCs can suppress fibroproliferation and inflammation during NAFLD resolution [278]. Resolving current discrepancies requires additional investigation to define the precise mechanisms through which DCs influence NAFLD progression (Table 2).

**Table 2:** Partial functional and metabolic changes of DC in non-oncological diseases

Diseases		Main DC subsets	Metabolic change	Functional change	Changed signal pathway
**Autoimmune Disease**	**RA** [201, 279]	cDC, moDC	Enhanced glycolysis; Reduced OXPHOS; Reduced FAO; Succinate increases	Increased secretion of inflammatory factors; Inducing an increase in Th1/17 differentiation	HIF-1α; JAK-STAT; PPARγ
	**SLE** [203, 279]	cDC, pDC	Disruptive mitochondrial metabolism; ROS overload; mtDNA damage	Inducing an increase in Th17 differentiation; pDC secrete more IFN	JAK-STAT; mTOR; cGAS-SING
**Allergic Diseases** [206, 210]		cDC, pDC	Reduced OXPHOS; Reduced FAO; May depend on the types of lipids and proteins in intestine; Sphingolipids homeostasis is disorder	Increased production of chemokines and inflammatory factors; Inducing B cell secretion of specific antibodies and T cell differentiation	PPARγ; mTOR; ERK-TIM-4; NF-κB
**Transplantation reaction** [214, 217]	**Rejection**	cDC	Metabolic changes initiated by ischemia–reperfusion injury; Enhanced sensitivity to ATP and adenosine	Towards Mature state; Enhanced migration to lymph nodes and antigen presentation ability; Release inflammatory factors; Enhanced T cell activation ability	HIF-1α; mTOR
	**Tolerance**	cDC	IDO expression upregulated, active tryptophan metabolism; Increased secretion of NO; Enhanced arginine metabolism	Towards semi-mature state; Inducing Treg differentiation; Inhibit T cell killing; High expression of immune checkpoint and anti-inflammatory factors	STAT3; NF-κB
**Infectious Disease**	**Bacteria** [10, 223]	cDC, moDC	Enhanced glycolysis driven by TLR; Reduced OXPHOS; Enhanced FAS; iNOS activation; Lipid accumulation and synthesis;	Enhanced antigen phagocytosis in the early stage and enhanced presentation ability in the later stage; Secreting inflammatory factors; Enhanced migration capability	AMPK; PI3K/Akt; mTOR; NF-κB
	**Viruses** [238, 248, 280]	pDC, cDC	Inflammation drives normal DC activation related metabolism; Damaged glycolysis and OXPHOS of pDC lead to antiviral immunosuppression;	Damaged metabolism leads to impaired secretion of IFN-I by pDC; Forming virus synapses, leading to the spread of the virus; Inducing immune checkpoint expression leads to immune tolerance	Ras/MAPK; JAK/STAT; PI3K/Akt; mTOR; β-catenin
	**Fungus** [251, 255, 281]	cDC, moDC	Enhanced glycolysis; Damaged OXPHOS of cDC lead to immune activation; Enhanced arginine metabolism; Enhanced FAO and FAS; Lipid accumulation; Damaged OXPHOS of moDC lead to immune inhibition;	Secreting inflammatory factors and inducing Th1/2/17 polarization to activate immune response;	PI3K/Akt; PPAR-γ; mTOR/SREBP; HIF-1α; NF-κB
	**Parasites** [256, 282]	cDC, moDC	Inducing mitochondrial synthesis to enhance OXPHOS and enhancing FAS to support DC activation; Enhancement of OXPHOS and inhibition of protein synthesis leads to immune tolerance; Increase ROS and NO production to kill pathogens	Promote the expression of immune checkpoints and inhibit the expression of co- stimulatory molecules; Inducing Treg differentiation; Hindering the maturity and migration of DC; Inducing Th1/2 differentiation and increasing secretion of pro-inflammatory cytokines	SOCS1/3; MAPK; PI3K; JAK-STAT; PPAR-γ; PGC-1α
**Chronic Inflammatory Diseases** [120, 272, 273]		cDC, pDC	Enhanced tryptophan metabolism to induce immune tolerance; During the progression of the disease, metabolic characteristics similar to activated DC; Retinoic acid synthesis is beneficial for the formation of tolerant DC	pDC intestinal infiltration increases; pDC co- stimulatory molecule expression and massive release of inflammatory factors; cDC show impaired ability to induce Treg differentiation, but it promotes the activation of Th1/2/17 and produces massive inflammatory factors	PLCβ-PKC; mTOR; AMPK; NF-κB; HIF-1α

## DCs and tumor immunity

Within the TME, DCs undergo profound functional dysregulation driven by immunosuppressive factors, hypoxic stress, and stromal interactions. These perturbations converge on metabolic reprogramming centered on acetyl-CoA flux, which orchestrates lipid metabolism, OXPHOS, and epigenetic modifications. Consequently, acetyl-CoA availability dictates DCs antigen presentation, migration, and cytokine secretion—pivotal processes for antitumor immunity. Elucidating acetyl-CoA-mediated metabolic-immune crosstalk unveils novel therapeutic strategies to reinvigorate DCs function and overcome tumor immune evasion.

### DCs dysfunction in the TME

#### Tumor derived factors interfere with DCs function

The TME consists of tumor cells, immune cells, stromal cells, cytokines, and microvasculature. Tumor-derived factors facilitate immune evasion by disrupting DCs function and inducing a tolerogenic phenotype, thereby enabling tumors to evade immune detection. TGF-β, produced by Tregs, fibroblasts, and macrophages, establishes an immunosuppressive TME that drives angiogenesis, metastasis, therapy resistance, and epithelial-mesenchymal transition [283]. This cytokine, along with PGE2, upregulates PD-L1 on DCs while stimulating IDO and CCL22 secretion, which promotes Treg differentiation and infiltration [284–286]. Pancreatic cancer models demonstrate that TGF-β/PD-L1 blockade enhances effector DCs activity [287], whereas TGF-β receptor knockdown in DCs boosts IFN-γ production and tumor cell clearance in cholangiocarcinoma models [288]. TGF-β inhibition also potentiates radiotherapy by improving DCs cytoskeletal reorganization, lymph node homing, and CTL activation in ovarian cancer [289]. Emerging evidence indicates that TGF-β disrupts DCs metabolism, compromising antigen processing and migration, with these effects amplified by the acidic TME [290]. Smad4, a critical TGF-β signaling component, restores tumor immunogenicity in Smad4-deficient cells, driving cDC1s activation and antigen uptake [291].

Under physiological conditions, insulin-like growth factor (IGF) stimulates protein synthesis and cell proliferation through ERK and Akt signaling [292]. However, in tumors, IGF impedes TADCs maturation, triggering IL-10 and TNF-α release, which disrupts DCs function and facilitates immune evasion [293]. IL-10, associated with poor clinical outcomes, suppresses DCs activity via JAK-STAT3 signaling, impairing antigen presentation, MHC-I expression, and DCs differentiation [294–298]. It also amplifies Treg-mediated suppression of T-cell activation [299, 300]. By downregulating pro-inflammatory cytokines (e.g., IFN-γ, TNF-α, IL-12) and CCL7, IL-10 further obstructs DCs migration [301, 302]. Neutralizing IL-10 in the TME may improve immunotherapy outcomes.

VEGF, induced by hypoxia via HIF-1, stimulates angiogenesis while inhibiting DCs function through VEGFR binding, which attenuates T-cell responses [303, 304]. VEGFR1 and VEGFR2 distinctly suppress DCs maturation and migration, respectively [305, 306]. Murine studies show that VEGF blockade synergizes with peptide-pulsed DCs to amplify antitumor immunity [307, 308], whereas in vitro, VEGF impedes monocyte differentiation into DCs [305, 309]. In contrast, DCs-derived VEGF-C, upregulated by IFN-γ, drives lymphangiogenesis and DCs maturation, aggravating tissue injury in transplantation and autoimmune disorders [310–312]. VEGF-C also augments DCs trafficking via CCL21, enhancing radiotherapy efficacy in meningioma [313]. These findings highlight the context-dependent duality of VEGF in DCs regulation, shaped by isoform and receptor specificity.

#### Tumor hypoxic environment and stromal cells disrupt DCs function

The TME demonstrates heightened lipid metabolism that sustains tumor proliferation and migration. Beyond fueling tumor growth, lipids actively suppress immune cell function, thereby accelerating tumor progression [314]. Polyunsaturated fatty acids (PUFAs) play a pivotal role, as their peroxidation yields lipid peroxides [315]. In DCs, PUFA phospholipids initiate COX- and LOX-mediated pathways that generate immunosuppressive mediators such as PGE2 [316]. Hypoxia reduces glutathione peroxidase 4 expression, compromising redox balance and potentially inducing ferroptosis [317, 318]. Ferroptosis-derived peroxidized lipids further suppress T-cell activity and hinder DCs antigen cross-presentation, exacerbating immune evasion [318, 319].

Hypoxic niches accumulate Tregs, M2 macrophages, and tolerogenic DCs [320]. Hypoxia regulates DCs function through ROS: moderate ROS levels stimulate DCs activation via p38-MAPK and ERK1/2 pathways, whereas excessive ROS promotes lipid peroxidation and ER stress, disrupting antigen presentation [107, 321]. TLR-activated DCs generate superoxide anions through NOX2, elevating endosomal pH and impairing antigen processing [322, 323]. While these observations underscore the need for deeper investigation of ROS mechanisms in TADCs, impaired antigen presentation appears more consequential than altered co-stimulatory molecule expression [324, 325]. Hypoxia-induced HIF activation also attenuates DCs migration by suppressing CCR7 and modulates cytokine secretion (e.g., VEGF, IL-12, IFN-γ), thereby inhibiting T-cell priming [326, 327]. Paradoxically, HIF may enhance DCs maturation during inflammation by upregulating glycolytic pathways [328]. Emerging evidence implicates hypoxia-driven DCs dysfunction in hepatocellular carcinoma via the SPP1-CD44 axis [329], warranting further mechanistic studies.

Cancer-associated fibroblasts (CAFs), a dominant stromal component, exhibit strong prognostic associations with disease outcomes [330]. Both CAFs and endothelial cells (ECs) critically modulate immune cell behavior. In liver cancer, activated hepatic stellate cells—the principal CAF precursors—orchestrate key immunosuppressive TME remodeling. CAF-derived IL-6 activates the STAT3 pathway in monocytes, inducing an inhibitory DCs phenotype characterized by downregulated CD86, CD40, and MHC-II [331, 332]. CAFs additionally secrete TGF-β and VEGF to further remodel the TME and support tumor advancement [332]. In lung cancer models, fibroblast-derived COX-2/PGE2 disrupts DCs function, fostering metastatic spread [333]. Col13a1^+^ CAFs preferentially recruit tumor-associated macrophages and Tregs while excluding DCs, thereby attenuating immune responses [334]. ECs form selective vascular barriers that facilitate Treg infiltration while restricting DCs, effector T cells, and NK cells [335, 336]. Through Stk11-Scf signaling, ECs govern DCs precursor differentiation and drive VEGF-mediated MAPK/ERK activation to induce endothelial-like DCs transformation [337, 338]. Notably, EC/DCs hybrid cells demonstrate superior T-cell stimulatory capacity and antitumor activity in preclinical models, suggesting therapeutic potential for targeting EC-DCs crosstalk [339, 340].

### Acetyl-CoA and DCs metabolism

Metabolic reprogramming, a process where cells dynamically adjust their metabolic activities in response to diverse stimuli, engages multiple interconnected metabolic pathways and plays a pivotal role in shaping disease progression [156, 341]. Within the TME, metabolic reprogramming serves as a cornerstone mechanism that profoundly influences the behavior of both tumor cells and immune cells. Among the various metabolic regulators, acetyl-CoA has emerged as a critical factor. Delving into the mechanisms of how acetyl-CoA modulates the metabolism of TADCs could unlock novel therapeutic targets, potentially enhancing the efficacy of TADCs in cancer treatment.

#### Lipid metabolism reprogramming

Lipid accumulation is a hallmark of TADCs, most notably through elevated triglyceride levels. While this lipid buildup substantially impairs DCs antigen processing capacity, its effect on antigen uptake remains limited. The influence on DCs co-stimulatory molecules and pro-inflammatory cytokines also appears modest [8, 342, 343]. TLR-mediated DCs activation often coincides with a metabolic shift from FAO to FAS, which enhances ER and Golgi function to support cytokine secretion and co-stimulatory molecule expression [3, 77, 201]. Two principal mechanisms drive lipid accumulation in TADCs: the endogenous pathway, potentially activated by SREBP transcription factors that upregulate genes encoding ACLY and FASN [344–346]. and the exogenous pathway, primarily mediated by increased fatty acid uptake through Msr [8], the exogenous pathway likely plays a more dominant role.

TADCs lipid accumulation produces distinct effects compared to normal DCs, potentially through synthesized fatty acids fueling OXPHOS to induce tolerogenic DCs phenotypes, while extracellular lipid uptake from tumor vesicles alters DCs signaling and organelle function [3, 8, 77, 182]. Combining tumor vaccines (Ad-survivin-transduced DCs or tyrosinase peptide-loaded DCs) with the ACC inhibitor TOFA improves therapeutic outcomes [8], possibly by increasing the proportion of DCs-NL (normal lipid DCs) within tumors. Although excessive fatty acid accumulation generally impairs DCs-mediated T cell activation, this effect varies with the TME, potentially explaining observed differences in DCs dysfunction across tumor types [347].

The balance between FAO and FAS also influences DCs differentiation, where etomoxir promotes cDC2s while suppressing cDC1s, contributing to immune dysfunction [13, 348]. In TADCs, nutrient competition may reduce acetyl-CoA availability, activating AMPK to enhance FAO and alter differentiation phenotypes [71, 349, 350], suggesting a mechanism by which acetyl-CoA modulates DCs anti-tumor function. Acetyl-CoA and lipid metabolism further regulate mTOR pathway activity: acetyl-CoA can activate mTORC1 via Raptor acetylation [351], whereas ACC-derived malonyl-CoA binds mTORC1 to inhibit Raptor [352]. This intricate crosstalk between acetyl-CoA, lipid metabolism, and mTOR may underlie the variable efficacy of targeting these pathways individually in cancer therapy.

#### Oxidative metabolism

Acetyl-CoA participates in the TCA cycle to produce NADH and FADH2 within mitochondria, subsequently fueling the electron transport chain for OXPHOS — the dominant ATP generation pathway in most cells [353]. Impaired OXPHOS in DCs correlates with diminished therapeutic efficacy of DCs vaccines in melanoma patients, establishing a direct link between DCs metabolic state and clinical outcomes [354, 355]. This metabolic impairment primarily stems from restricted acetyl-CoA influx, with OXPHOS efficiency being governed by mitochondrial integrity through mammalian sterile 20-like kinase (Mst) 1/2 regulation [72]. Tumor-mediated Mst signaling disrupts DCs mitochondrial function, suppressing OXPHOS and consequently impairing T-cell activation via the iNOS pathway [79]. Paradoxically, Mst deficiency enhances DCs development in vitro, likely through mitochondrial destabilization that reduces OXPHOS during DCs differentiation [356]. The Mst1/2 pathway appears crucial for acetyl-CoA-mediated OXPHOS regulation, potentially by modulating endogenous acetyl-CoA synthesis in DCs.

Tumors create nutrient competition by aggressively consuming oxygen and resources from their microenvironment [79]. Hypoxia triggers DCs to upregulate HIF-1α, which shifts metabolism toward glycolysis while suppressing OXPHOS [78, 79] and stimulating pro-inflammatory cytokine secretion (TNF-α, IL-6, IL-1β) [357, 358]. However, sustained HIF-1α activation in TADCs induces immune tolerance [359]. This transcription factor inhibits pyruvate dehydrogenase via pyruvate dehydrogenase kinase 1, while its own expression is conversely regulated by acetyl-CoA-dependent histone acetylation at HIF-1α target gene promoters [360, 361]. This reciprocal regulation between HIF-1α and acetyl-CoA suggests a metabolic checkpoint for DCs function that could inform therapeutic strategies.

OXPHOS restoration in DCs enhances immunotherapy responses [355], positioning acetyl-CoA modulation as a promising intervention. However, excessive OXPHOS or FAO risks ROS accumulation that triggers lipid peroxidation, ER stress, and antigen presentation defects [106, 184]. IRE1α-XBP1 activation further disrupts DCs metabolism by driving lipogenesis [106, 362] and inducing DNA damage-mediated apoptosis [104]. TADCs accumulating oxidized lipids exhibit compromised antigen presentation capacity, highlighting the need for precise OXPHOS modulation when using acetyl-CoA to enhance DCs function. Furthermore, the dependence of different DCs subpopulations on OXPHOS exhibited variation. And AMPK activation-driven enhancement of OXPHOS frequently results in the formation of a DCs tolerance phenotype. This further underscore the necessity for further exploration of the effects of acetyl-CoA-driven OXPHOS enhancement on DCs phenotypes under different spatiotemporal localizations.

### Acetyl-CoA modulates DCs-mediated antitumor immunity response

The anti-tumor immune function of DCs depends primarily on their ability to present antigens efficiently, migrate to lymph nodes and regulate the immune system through cytokines. These critical functions are profoundly influenced by metabolic reprogramming in the TME, particularly by fluctuations in acetyl-CoA levels. As a key metabolic intermediate and substrate for epigenetic modifications (e.g. histone acetylation), acetyl-CoA regulates the expression of MHC and co-stimulatory molecules, chemokine receptor-mediated migration and the secretion of various key cytokines. Therefore, disruptions to acetyl-CoA metabolism within the TME that interfere with these mechanisms constitute a fundamental basis for DCs dysfunction and tumor immune escape.

#### Antigen presentation and activation

DCs mediate tumor immunity by presenting antigens via MHC molecules, enabling CD8^+^ T cells to recognize and eliminate tumor cells [363]. Growing evidence highlights the importance of CD4^+^ T cell responses triggered by MHC-II in antitumor immunity [364]. Acetyl-CoA modulates MHC gene expression through its involvement in regulatory acetylation events. MHC-II gene expression depends on acetylation dynamics governed by histone acetyltransferases (HATs) and histone deacetylases (HDACs). The master regulator CIITA drives MHC-II transcription through histone acetylation, binding the MHC-II promoter with other transcription factors to acetylate H3 and H4 histones [9]. Acetyl-CoA may thus enhance MHC-II expression by facilitating acetylation of transcriptional activators. Histone H4 acetylation also activates the enhancer A region of MHC-I genes [365]. The transcriptional co-activator p300/CBP maintains MHC-I expression, while its associated factor PCAF counteracts HDAC activity [366]. Metabolic reprogramming in TADCs alters acetyl-CoA availability, potentially impairing MHC expression and antigen presentation to promote immunosuppression. Although cellular acetyl-CoA exhibits compartmentalized distribution [7], elevated FAS and glucose deprivation in TADCs may deplete nuclear acetyl-CoA pools, disrupting expression of MHC-like genes.

CD80/86 serve as essential DCs co-stimulatory molecules, with mitochondrial p32 regulating acetyl-CoA flux into the TCA cycle to sustain citrate levels required for their expression [367]. Lipid mediators like PGE2 modulate both co-stimulatory molecule expression and DCs maturation by suppressing NK cell activity. Tumor-derived PGE2 inhibits DCs maturation and DAMP uptake, consequently impairing T cell activation [368, 369]. COX inhibition can restore DCs immune function [368]. Since prostaglandin synthesis from phospholipids requires acetyl-CoA, recent studies have investigated the interplay between acetyl-CoA and prostaglandin metabolism [370].

#### DCs migration

DCs must present antigen in lymph nodes to fully activate T cells before these effector cells can migrate to and attack tumors [371, 372]. CCR7 and its ligand CCL21 mediate DCs migration through lymphatic vessels, with inflammatory cytokines further facilitating this process [373–375]. Melanoma patients exhibiting low CCR7 expression show poorer clinical outcomes, underscoring this chemokine's prognostic significance [376]. Cellular metabolism directly regulates CCR7 expression in DCs, as demonstrated by glycolysis-dependent promotion of CCR7 oligomerization and subsequent effects on migratory capacity [83]. Cholesterol metabolism also influences DCs function in immune disorders, where balanced levels stabilize MHC/antigen complexes while excess cholesterol impairs lymph node homing [377–379]. Lipid composition changes during DCs maturation further modulate migration dynamics [380]. While acetyl-CoA's metabolic control over these pathways is established, its specific relationship with DCs migratory behavior in tumors remains an active research focus. Epigenetic regulation through methylation and acetylation additionally governs chemokine expression, though the exact connection to acetyl-CoA requires clarification [376, 381].

Tumor cells suppress DCs recruitment by inhibiting CCL4 and CCL5 secretion via β-catenin pathway activation [103, 382]. In glioblastoma, HMGCL knockdown decreases acetyl-CoA levels and consequently suppresses β-catenin signaling [383]. Aberrant β-catenin activation in TADCs appears to promote immune tolerance, suggesting a mechanistic link between acetyl-CoA metabolism and impaired DCs function [103, 384]. These findings collectively advance our understanding of how acetyl-CoA influences DCs migration and antitumor activity.

#### Cytokines

Primary T cells depend on persistent MHC molecule stimulation and intermittent IL-7 signaling to sustain long-term survival while preserving homeostasis and functionality [385–387]. TSC1-deficient DCs exhibit reduced T cell activation due to compromised IL-7 secretion [87, 344]. Stromal cells and DCs produce IL-7, a cytokine increasingly employed in T-cell therapies [388–390]. Through STAT5 and mTOR pathway activation, IL-7 induces histone acetylation at the II9 promoter, governing Th9 cell differentiation and antitumor responses [391], with acetyl-CoA serving as a crucial mediator of mTOR-dependent acetylation regulation [87]. These findings suggest acetyl-CoA may influence DCs function both directly and through DCs-mediated modulation of other immune cells.

The PI3K/Akt/mTOR pathway becomes activated in TME cells following ACSS2 upregulation, the primary enzyme for acetyl-CoA production [168, 392–394]. Since both ACSS2 and mTOR regulate DCs cytokine secretion, with acetyl-CoA mediating this effect, an ACSS2-mTOR axis likely governs TADCs functionality. Additionally, DCs suppress CD8^+^ T cell activation through mTOR-dependent β-catenin signaling that drives IL-10 secretion [3, 87].

**Fig. 4:**
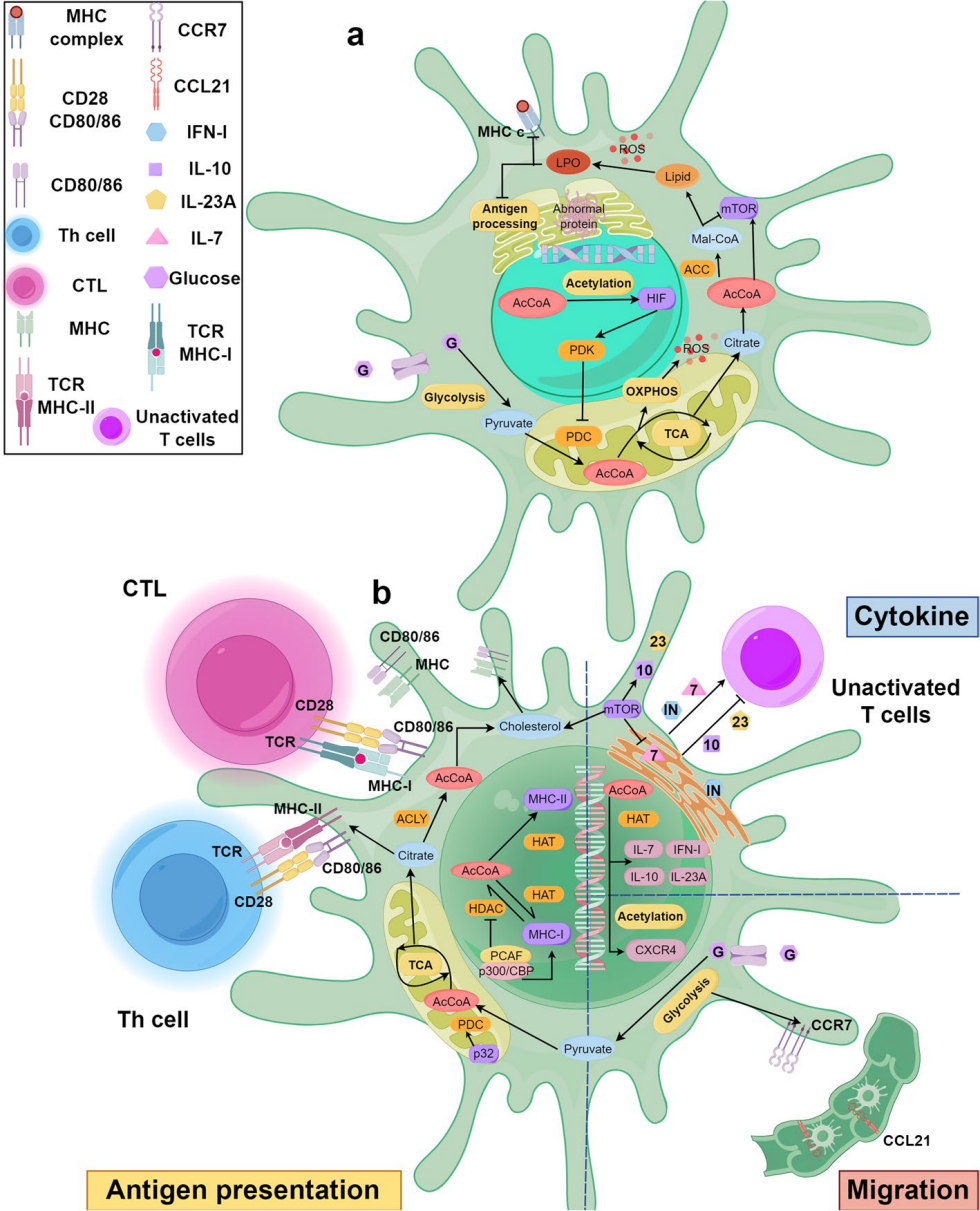
The effect of acetyl-CoA on DC. **a** Acetyl CoA and DC metabolism. Pyruvate produced by DC glycolysis is converted to acetyl-CoA to enter TCA cycle, catalyzed by PDC and transported by MPC. Acetyl-CoA regulates the expression of the HIF gene, which inhibits PDC activity via PDK, leading to impaired glycolysis. Acetyl-CoA prepares for oxidative phosphorylation through the TCA cycle, which generates ROS. The accumulation of ROS leads to lipid peroxidation and ER stress, impairing antigen processing and presentation. The lipid synthesis of DCs is also initiated by acetyl-CoA, which is catalyzed by the ACC to produce Mal-CoA. Acetyl-CoA can regulate the activity of mTOR to further influence the overall metabolic phenotype of the cell. Mal-CoA inhibits the mTOR pathway by suppressing Raptor. Mal-CoA also synthesizes lipids, which may contribute to the accumulation of oxidized lipids that interfere with the immune response. **b** Acetyl CoA and DC function. In the nucleus, acetyl-CoA affects histone acetylation, which controls the expression of genes involved in immune function. Outside the nucleus, glycolysis occurs with CCR7 oligomerization. Citric acid and cholesterol affect CD80/86 expression and MHC stability. The above processes may be regulated by mTOR, affecting DC functions such as antigen presentation, cytokine secretion and migration. This suggests a link between acetyl-CoA and DC function. PDK, pyruvate dehydrogenase kinase

pDCs and certain cDC2s subsets produce IFN-I to upregulate DCs MHC expression, enhancing antigen presentation while promoting cDC1s migration and activation [395]. Yet TME inhibit pDCs IFN-I secretion, potentially through TGF-β and other suppressive factors. As tumor-derived TGF-β levels correlate with acetyl-CoA concentrations [56, 396], and given the established link between cellular metabolism and IFN production [157, 397], acetyl-CoA-mediated epigenetic regulation of IFN loci likely contributes to functional differences among tumor-associated DCs subsets (Fig. 4).

## Therapeutic potential and clinical applications

Given the critical role of metabolism in TADCs function, modulating TADCs metabolism has emerged as a promising strategy to enhance tumor immunotherapy efficacy. DCs vaccines represent the most widely used DCs-based treatment, offering relatively mild side effects compared to other approaches. Nevertheless, functional limitations persist, particularly when DCs encounter tumor-associated signals in vivo, often leading to impaired activity. This dysfunction stems from intricate cellular interactions within the TME. Acetyl-CoA not only regulates TADCs function but also influences T-cell activity and tumor cell behavior. Consequently, therapeutic targeting of acetyl-CoA metabolism may provide an innovative approach to improve DCs immunotherapy outcomes.

### Acetyl-CoA-targeting strategies

The efficacy of DCs vaccines depends critically on successful tumor antigen loading, and tumor cell lysates effectively enhance DCs-mediated T cell activation [398–400]. Acetyl-CoA regulates autophagy through acetylation, modulating both autophagy gene transcription and protein modifications [401]. In colon cancer, overexpression of PTEN-inducible kinase 1 depletes acetyl-CoA and induces tumor cell autophagy [159]. The mTOR and AMPK pathways respond to acetyl-CoA fluctuations, thereby regulating autophagy initiation [170]. This acetyl-CoA-mediated autophagic mechanism may improve DCs therapy outcomes.

Acetyl-CoA directly enhances DCs antitumor activity by modulating IL-12 production, a pivotal cytokine for DCs vaccine efficacy [402]. While ACC inhibitors suppress IL-12 secretion [403], TOFA synergizes with DCs vaccines to improve therapeutic outcomes[8], suggesting DCs maturation state influences these metabolic interventions. TLR3 agonists like ARNAX induce antitumor immunity in DCs through cytokine-independent mechanisms [404]. Although poly I:C-activated TLR3 triggers acetyl-CoA-dependent acetylation in endothelial cells [405], its role in DCs remains underexplored and warrants further investigation.

Metabolic heterogeneity among DCs subsets suggests that acetyl-CoA manipulation could steer in vitro differentiation toward more effective vaccine phenotypes. CD8α^+^ DCs rely heavily on oxidative metabolism and dominate IL-12 production, enabling robust CD8^+^ T cell activation [72, 406]. As discussed earlier, acetyl-CoA promotes this oxidative phenotype, implying its utility in engineering DCs for enhanced antitumor immunity. Acetyl-CoA further intersects with PD-1 signaling—maintaining c-Myc acetylation to upregulate PD-L1 in NSCLC while PD-1 suppresses T cell proliferation via ACLY inhibition [407, 408]. In PD-1-deficient models, elevated mitochondrial metabolites like cholesterol drive DCs inflammatory polarization and antigen presentation [400, 409], supporting combined acetyl-CoA modulation with DCs vaccines to potentiate immune checkpoint inhibition. Immune checkpoint silencing in DCs augments their T cell stimulatory capacity [410, 411]. Further combination of such modified DCs may be able to increase the sensitivity of tumor cells to ICI treatment while avoiding checkpoint inhibition of DCs vaccines.

Acetyl-CoA-metabolizing enzymes also contribute directly to tumorigenesis [412, 413], prompting clinical development of inhibitors targeting ACC (PF-05221304), ACSS2 (MTB-9655), and ACLY (SB-204990179) [414–418] (Table 3).

**Table 3:** Application of Acetyl-CoA related targets in cancer therapy

Target	Molecule	Cancer	Status
**ACC**	TOFA [419–421]	ESCC, Breast cancer, OV	Preclinical
	ND-646 [422, 423]	CRC, PAAD, Lung cancer	Preclinical
	ND-654 [424]	HCC	Preclinical
	phenoxy-phenyl isoxazoles [425]	Breast cancer	Preclinical
	ND-630 [426]	PRAD	Preclinical
	Bezafibrate [427, 428]	NSCLC	Phase I trial (UMIN000017854)
	Soraphen A [429]	Breast cancer	Preclinical
	Bezafibrate [428]	Breast cancer	Preclinical
**ACLY**	BMS-303141 [418]	HCC	Preclinical
	Hydroxycitrate [430]	PRAD, SKCM, Lung cancer	Preclinical
	SB-204990 [431]	Thyroid cancer	Preclinical
	Bempedoic acid/ETC-1002 [432]	HCC	Preclinical
	Cucurbitacin B [433]	PRAD	Preclinical
	Emodin derivatives [434]	Lung cancer	Preclinical
	HCA[435]	PAAD	Preclinical
	NDI-091143[431]	Thyroid cancer	Preclinical
**PDC**	CPI-163 [436]	Leukemia	Phase I trial
	DCA [437, 438]	Brain tumor	Phase I trial (NCT01111097)
		Soid tumor	Phase I trial
**HMGCR**	Statin [439, 440]	Head and neck cancer	Approved
		PRAD	Preclinical
**ACSS2**	VY-3–249 [441]	Breast cancer	Preclinical
	AD-8007 [442]	Brain metastases	Preclinical
**KAT**	WM-1119 [443]	Lymphoma	Preclinical
	PF-07248144 [444]	Breast cancer	Phase I trial (NCT04606446)
	CCS1477 [445]	PRAD	Phase I/II trial (NCT03568656)
	FT-6876 [446]	Breast cancer	Preclinical
	FT-7051 [446]	PRAD	Phase I trial (NCT04575766)
	Curcumin [447]	CRC	Preclinical
	WM-3855 [448]	PRAD	Preclinical
	MG149 [449]	Gallbladder cancer	Preclinical
	ICG-001 [450]	Endometrial cancer	Preclinical
**HDAC**	Vorinostat [451]	Cervical cancer	Approved
	Resminostat [452]	PAAD	Approved
	Romidepsin [453]	Lymphoma	Phase I/II trial (NCT02783625)
	Panobinostat [454]	Myeloma	Phase III trial
	Belinostat [455]	PTCL	Approved
	LMK-235 [456]	PAAD	Preclinical
	Ricolinostat [457]	Breast cancer	Phase I trial (NCT02632071)
	Chidamide [458]	CRC	Phase II trial (NCT04724239)
	Tucidinostat [459]	PTCL	Phase II trial (NCT02953652)
	Entinostat [460]	NSCLC	Phase I trial (NCT04631029)
**FASN**	Orlistat [461]	CRC	Preclinical
	TVB-2640 [462]	HCC	Preclinical
	Cerulenin [463]	Cervical cancer	Preclinical
	C75 [464]	Breast cancer	Preclinical
	Omeprazole [465]	PRAD	Phase II trial (NCT04337580)
**CPT**	Etomoxir [466]	Breast cancer, Leukemia	Preclinical
	St1326 [467]	Lymphoma	Preclinical
	Perhexiline [468]	Glioblastoma	Preclinical
**CRAT**	Mildronate [469]	HCC	Preclinical
**SIRT1**	Resveratrol [470]	Cervical cancer	Preclinical
**GLS**	CB-839 [471]	Breast cancer	Preclinical
	DRP-104 [472]	PAAD	Preclinical
	IACS-6274 [473, 474]	NSCLC	Preclinical

*ACC* Acetyl-CoA carboxylase, *ACLY* ATP citrate lyase, *ACSS2* Acetyl-CoA synthetase 2, *BRCA CPT* Carnitine palmitoyltransferase, *CRC* Colorectal cance, *CRAT* Carnitine acetyltransferase, *ESCC* Esophageal squamous cell carcinoma, *FASN* fatty acid synthase, *GLS* Glutaminase, *HCC* Hepatocellular carcinoma, *HDAC* Histone deacetylases, *HMGCR* 3-hydroxy-3- methylglutaryl-CoA reductase, *KAT* Lysine acetyltransferase, *NSCLC* Non-small cell lung cancer, *OV* Ovarian carcinoma, *PAAD* Pancreatic adenocarcinoma, *PDC* pyruvate dehydrogenase complex, *PRAD* Prostate adenocarcinoma, *PTCL* Peripheral T cell lymphomas, *SIRT1* Sirtuin 1, *SKCM* skin cutaneous melanoma

**Fig. 5:**
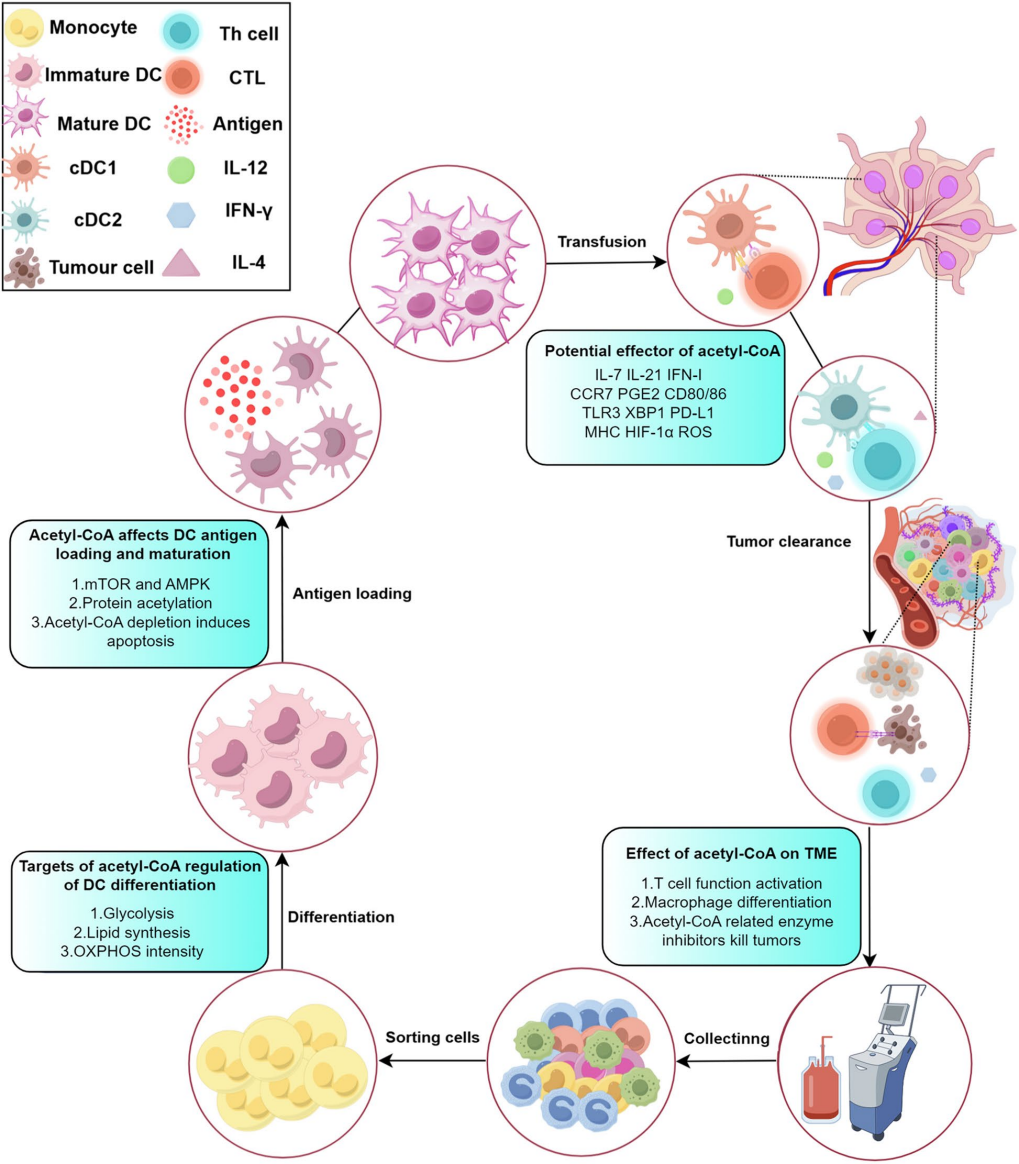
The therapeutic potential of acetyl-CoA. DC vaccines are the most widely used DC-based immunotherapy. By collecting blood from patients and sorting the cells, we can induce differentiation and maturation using activating factors. Acetyl-CoA may target the metabolism to control differentiation and maturation, and modulating this process with acetyl-CoA may further promote the generation of more effector phenotypes of DC. Co-culturing tumor antigens with activated DCs to load them with antigens and using acetyl-CoA to modulate tumor autophagy and DC phagocytosis may help tum our vaccines work better. The efficacy of antigen-loaded DCs re-infused into the patient depends on T cells being activated by molecules like IL-12 and CD80/86. The effect of acetyl-CoA on these molecules shows promise in improving DC efficacy. Acetyl-CoA affects other cells in the TME, making it a fully integrated strategy for therapeutic development

In conclusion, acetyl-CoA may regulate DCs and DCs-associated cells through multi-omics mechanisms at metabolic and genetic levels, potentially enhancing the therapeutic efficacy of DCs-based treatments. However, further studies are required to validate its clinical applicability and broader potential (Fig. 5).

### DCs metabolic engineering

Targeting lipid metabolism with TOFA and FASN inhibitors like cerulenin restored TADCs immunological activity across multiple tumor types, while simultaneously reducing aberrant lipid accumulation [8, 475]. The XBP1 pathway not only drives endogenous FAS in TADCs but also mediates ER stress, with polyethyleneimine-siRNA nanocomposites targeting IRE1α-XBP1 effectively suppressing lipid accumulation and boosting TADCs immunostimulatory function [104]. The in situ nanovaccine TPOP demonstrated dual efficacy in modulating lipid metabolism and activating innate immunity, thereby improving TADCs cross-presentation [476]. Another nanoparticle, TS-PP@FU, binds TADCs Msr1 to deliver triple metabolic inhibition—blocking exogenous lipid uptake, endogenous synthesis, and lipid-related gene transcription—which restored TADCs antitumor capacity in cytokine secretion and CTL activation [102]. Notably, combining these lipid-targeting approaches with ICI therapy substantially enhanced treatment efficacy [8, 102, 476].

PGE2-mediated TADCs suppression was reversed by COX-2 inhibition with NS-398, while aspirin and celecoxib improved anti-PD-1 responses in melanoma [477, 478]. EP4 receptor antagonists AAT-008 and E7046 increased cDC1s frequency and CXCL10-dependent CTL recruitment respectively, with E7046 synergizing with anti-CTLA-4 in colon cancer models [479].

Tryptophan metabolism similarly impairs tumor-infiltrating immune cells, where tumor-secreted IDO inhibits TADCs function. Clinical-stage IDO inhibitors like epacadostat enhanced DCs cytokine production while suppressing Tregs [480], whereas indoximod potentiated DCs vaccine responses by downregulating IDO via AhR signaling [481, 482].

### Combination therapies

Combination therapies demonstrate significant efficacy in cancer treatment, particularly immune checkpoint blockade and its integration with conventional radiotherapy or chemotherapy. Integrating DCs therapy with chemotherapy, adjuvants, or ICI improves outcomes while reducing adverse effects [483]. DCs metabolism directly influences immune checkpoint expression and activation, with PD-L1, CD40, and TIM-3 levels modulated by the interplay between mitochondrial metabolism and glycolysis in DCs subsets. TME-derived lactate suppresses both OXPHOS and glycolysis in DCs via monocarboxylate transporters, impairing DCs maturation and subsequent T cell activation. Concurrent metabolic inhibition with DCs vaccines and ICI therapy could restore antitumor responses and potentiate immune checkpoint blockade [4]. Pretreating DCs vaccines with metabolic inhibitors or administering these agents in vivo may heighten patient responsiveness to ICI and chemotherapy. Emerging evidence suggests mTOR inhibitors amplify tumor vaccine efficacy through both systemic delivery and ex vivo DCs treatment [88, 484]. While chemotherapy triggers immunogenic cell death and promotes DCs maturation, the scarcity of Ly6c^+^CD103^+^ DCs—critical for antitumor immunity—limits its effectiveness. BTK-IDO axis inhibition attenuates tryptophan metabolism while stimulating differentiation of these DCs subsets, potentially augmenting chemotherapy responses [485]. Paradoxically, chemotherapy elevates IDO expression in DCs; 1-MT-mediated IDO inhibition reduces Treg infiltration, enhances antitumor immunity, and improves survival [486]. Metabolic checkpoint targeting in DCs could optimize vaccine resistance to the TME, serving as a pivotal adjuvant strategy when combined with radiotherapy, chemotherapy, ICI, or CAR-T therapies. Restructuring DCs and global tumor metabolism may prove essential for overcoming immunosuppression. In particular, modifications that target the immunosuppressive crosstalk between dendritic cells and T cells may hold the key to achieving a significant improvement in patient survival [487]. Preclinical data indicate metabolic checkpoint modulation generates an immune-permissive TME, potentially improving CAR-T/CAR-NK cell therapy efficacy and ICI sensitivity.

## Conclusion

DCs are crucial for antitumor immunity, but their dysfunction in the TME limits immunotherapy efficacy. This review examines DCs ontogeny, subset diversity, and metabolic flexibility, with a focus on acetyl-CoA as a key metabolic regulator of immunometabolism reprogramming. We analyze how acetyl-CoA regulates lipid synthesis, mitochondrial dynamics, and epigenetic changes in DCs, linking these processes to antigen presentation, T cell activation, and migration deficits in tumors. Additionally, we explore different principles of DCs dysfunction in infections, autoimmune diseases, and chronic inflammation to achieve comprehensive recognition of DCs functional state changes.

Acetyl-CoA directs DCs functional fate through three interconnected pathways. In lipid metabolism, TADCs exhibit pathological lipid accumulation from disordered FAS and Msr-mediated uptake. This overload impairs antigen cross-presentation via ER stress and oxidized lipid-induced MHC-peptide destabilization. ACC or FAO inhibition restores DCs immune activity and enhances ICI-induced T cell infiltration. Acetyl-CoA-driven FAS normally supports DCs activation but becomes pathological in the TME, possibly due to altered lipid species and localization. DCs subset-specific transcriptional regulation of metabolic enzymes may further dictate lipid responses. Epigenetically, acetyl-CoA regulates histone acetylation at immunogenic loci via KATs. TADCs hypoacetylation, caused by acetyl-CoA depletion or NO-mediated KAT inhibition, may silence immune activation genes. HDAC inhibitors or acetyl-CoA supplementation could be potential treatments, though outcome may be limited by incomplete understanding of acetylation dynamics and DCs subset heterogeneity. In mitochondrial dynamics, acetyl-CoA supports OXPHOS for DCs migration and cDC1s-mediated IL-12 secretion/CD8^+^ T cell activation. The TME may disrupt this via hypoxia/NO-induced ETC inhibition and HIF-1α-driven tolerance. However, OXPHOS effects are condition-dependent, with excessive FAO-driven OXPHOS potentially causing ROS-mediated lipid peroxidation. Precise modulation based on DCs state and microenvironmental cues is essential.

Translational insights underscore the potential of acetyl-CoA-centric therapies. Metabolic checkpoint targeting via ACLY or ACC inhibition reduces tumor lipids and enhances DCs antigen presentation. Advanced nanoformulations enable co-delivery of metabolic inhibitors with immune agonists, reprogramming TADCs and reversing checkpoint-driven exhaustion. In the future, it may become feasible to achieve local remodeling of DCs metabolism by further developing pH/ROS-responsive vectors for precise release of metabolic checkpoint inhibitors or acetyl-CoA derivatives. This approach could facilitate precise disease management and address the necessity for adaptation to the internal environment. Combination strategies integrating acetyl-CoA pathway modulation with DCs vaccines may be helpful to combat functional impairment in TME. Thus, metabolic checkpoint-modified DCs vaccines are expected to be a novel adjuvant therapy. Strategic integration with radiotherapy, chemotherapy, or ICI may enhance antitumor efficacy through broad immune activation.

Metabolic regulation, particularly through metabolic pathways integrated by acetyl-CoA, is a key signal that shapes DCs function. Exploring spatiotemporal dynamics of acetyl-CoA metabolism in DCs can link metabolism to immunity, enhancing immunotherapy approaches. The acetyl-CoA-DCs axis might offer new routes for immunometabolism therapies, helping remodel the TME and turn “cold” tumors “hot”.

## Data Availability

No applicable.
